# Novel Combination of Surface Markers for the Reliable and Comprehensive Identification of Human Thymic Epithelial Cells by Flow Cytometry: Quantitation and Transcriptional Characterization of Thymic Stroma in a Pediatric Cohort

**DOI:** 10.3389/fimmu.2021.740047

**Published:** 2021-09-30

**Authors:** Veronika Haunerdinger, Maria Domenica Moccia, Lennart Opitz, Stefano Vavassori, Hitendu Dave, Mathias M. Hauri-Hohl

**Affiliations:** ^1^ Division of Stem Cell Transplantation and Children’s Research Center, University Children’s Hospital, Zurich, Switzerland; ^2^ Functional Genomics Center Zurich, Swiss Federal Institute of Technology and University of Zurich, Zurich, Switzerland; ^3^ Division of Immunology and Children’s Research Center, University Children’s Hospital, Pediatric Immunology, Zurich, Switzerland; ^4^ Division of Congenital Cardiovascular Surgery, University Children’s Hospital and Children’s Research Centre, Zurich, Switzerland

**Keywords:** human thymus, thymic epithelial cells, podoplanin, CD49f, CD200, extracellular matrix, flow cytometry

## Abstract

Thymic epithelial cells (TECs) are essential in supporting the development of mature T cells from hematopoietic progenitor cells and facilitate their lineage-commitment, proliferation, T-cell receptor repertoire selection and maturation. While animal model systems have greatly aided in elucidating the contribution of stromal cells to these intricate processes, human tissue has been more difficult to study, partly due to a lack of suitable surface markers comprehensively defining human TECs. Here, we conducted a flow cytometry based surface marker screen to reliably identify and quantify human TECs and delineate medullary from cortical subsets. These findings were validated by transcriptomic and histologic means. The combination of EpCAM, podoplanin (pdpn), CD49f and CD200 comprehensively identified human TECs and not only allowed their reliable distinction in medullary and cortical subsets but also their detailed quantitation. Transcriptomic profiling of each subset in comparison to fibroblasts and endothelial cells confirmed the identity of the different stromal cell subsets sorted according to the proposed strategy. Our dataset not only demonstrated transcriptional similarities between TEC and cells of mesenchymal origin but furthermore revealed a subset-specific distribution of a specific set of extracellular matrix-related genes in TECs. This indicates that TECs significantly contribute to the distinct compartmentalization – and thus function – of the human thymus. We applied the strategy to quantify TEC subsets in 31 immunologically healthy children, which revealed sex-specific differences of TEC composition early in life. As the distribution of mature CD4- or CD8-single-positive thymocytes was correspondingly altered, the composition of the thymic epithelial compartment may directly impact on the CD4-CD8-lineage choice of thymocytes. We prove that the plain, reliable strategy proposed here to comprehensively identify human TEC subpopulations by flow cytometry based on surface marker expression is suitable to determine their frequency and phenotype in health and disease and allows sorting of live cells for downstream analysis. Its use reaches from a reliable diagnostic tool for thymic biopsies to improved phenotypic characterization of thymic grafts intended for therapeutic use.

## Introduction

The thymus provides the dedicated environment for the generation of mature, self-tolerant T cells from multipotent hematopoietic progenitors. In contrast to secondary lymphoid organs such as lymph nodes, which are devoid of epithelial cells, thymic function relies on thymic epithelial cells (TECs) (1, 2). Murine studies have been instrumental to our current understanding of TECs and their central role in catalyzing T-cell development. Cortical TECs (cTECs) provide the signals for T-lineage commitment, expansion of precursors and shaping positive selection of T cell receptor specificities (3, 4). Expressing tissue-restricted antigens (TRAs), medullary TECs (mTECs) contribute to the elimination of potentially auto-reactive clones, alternatively promote their conversion into regulatory T cells (1, 2) and support the functional maturation of post-selection T cells (5).

Throughout the thymus, a complex network of non-hematopoietic and hematopoietic accessory cells (including fibroblasts, endothelial cells, dendritic cells and macrophages) is organized within the two thymic compartments with dedicated functions (6). Those cells share tasks linked to the generation of T cells (7, 8), but also support the differentiation and maintenance of TECs (9, 10). In addition, TECs also rely on thymocytes at varying stages of their development for stimulatory crosstalk (11).

Due to their central role in thymic function, deficiencies affecting the development TECs or resulting in insufficient cues for their proliferation or maintenance from other thymic cells, result in qualitative or quantitative thymic dysfunction (12).

Though often neglected in cell-oriented studies, the extracellular matrix (ECM) is fundamentally involved in fostering cellular processes such as adhesion, migration, lineage commitment and cell-cell interactions. Correspondingly, it contributes to the formation and maintenance of specialized compartments within organs (13–15).

The use of single-cell RNA-sequencing in murine and human studies has recently revealed a great stromal cell heterogeneity, in particular within the medullary TEC compartment, whereas cTECs display seemingly less diversity on the level of gene expression (16–19). Despite some analogy between murine and human thymic stromal subsets with respect to their developmental dynamics (20, 21) and transcriptional profiles (16, 18), species-specific peculiarities are discernible. A notable example is the uniform expression of EpCAM on murine TEC, whereas human TEC subsets may demonstrate differential expression of this molecule (22). Thus, a profound understanding of the human thymus based on the detailed phenotyping and quantitation of stromal cells is required. Flow cytometry (FC) has proven indispensable for this purpose in murine studies and has led to the identification of distinct subpopulations of thymic stromal cells (2). Mainly due to the relative lack of corresponding markers suitable for FC in humans, histological analysis remains the cornerstone of thymus diagnostics and research in humans (23, 24).

Here, we performed a FC-based surface marker screening assay to identify a suitable set of surface molecules which are able to A) differentiate TECs from other CD45^-^ stromal cells and B) reliably delineate cTECs and mTECs. We identified high expression of podoplanin (pdpn) as a hallmark of human TECs, which in combination with EpCAM allows their unequivocal identification from other stromal cell subsets, while expression of CD49f and CD200 differentiate reliably between cTECs and mTECs. Transcriptomic analysis of bulk-sorted stromal cell subsets confirmed the proposed identification strategy, which was then used for the precise and comprehensive quantification of TECs in a large set of thymi derived from 31 immunologically healthy pediatric subjects. We found significant sex-specific differences in TEC and thymocyte subsets in the first three months postnatally. In essence, we provide a novel identification strategy for human TEC subsets, allowing for their comprehensive identification and quantitation.

## Material and Methods

### Human Thymic Tissue

Thymic tissue from 53 immunologically healthy children aged between 1 day and 11 years was obtained from children undergoing corrective cardiac surgery at the University Children’s Hospital Zurich. In addition, 2 thymi from patients with immunological phenotypes were obtained: 1 patient with myasthenia gravis (15 years old, female, high-dose steroid treatment) and 1 patient with 22q11.2 microdeletion syndrome (14 days old, female, no medication with known influence on thymic cell populations). All tissue was obtained in accordance with the declaration of Helsinki and in adherence to guidelines from the local ethical commission (No. 2017-00312). In total, 55 thymi were used in this study.

Thymic tissue was stored in phosphate-buffered saline (PBS) and processed within four hours. For thymocyte preparations, a small fragment of cleaned thymic tissue was placed between two pieces of 100 μm nylon mesh, and carefully rubbed with the bottom of a syringe. To obtain stromal cells, fragments were digested in PBS with Liberase TM 0.2 mg/ml (Roche) and DNAse I 0.02 mg/ml (Roche) at 37°C under repeated pipetting for 35minutes and filtered through a 100 µm nylon mesh.

### Enrichment of Antigen-Presenting Cells

Antigen presenting cells (APCs) were enriched byPercoll density gradient (25) centrifugation. In brief, a Percoll (GE Healthcare) solution (final density 1.07 g/ml with physiological osmolarity) was distributed to 2 centrifuge tubes, mixed with 25x10^8^ cells, overlayed with cold buffer (AutoMACS Running buffer, Miltenyi), and centrifuged at 3500xg for 20 minutes at 4°C with decreased acceleration and deceleration.

### Flow Cytometry Surface Staining

Samples were stained with fixable viability dye eFluor506 (BioLegend) The following fluorochrome-conjugated antibodies were used: BioLegend: CD11b (M1/70), CD155 (SKIL4), CD165 (SN2(N6-D11), CD1a (HI149), CD200 (OX-104), CD205 (HD-30), CD27 (O323), CD3 (HIT3a), CD31 (WM59), CD4 (OKT4), CD45 (2D1), CD49e (MKI-SAM-1), CD49f (GoH3), E-Cadherin (67A4), EpCAM (9C4), HLA-DR (L243), podoplanin (NC-08); Miltenyi: CD11c (REA618), CD8 (REA734). UEA-1 (Sigma-Aldrich L9006) was used at 100 μg/ml. Samples were fixed with Fixation Buffer (BioLegend 420801).

### Flow Cytometry Intracellular Staining

The Foxp3/transcription buffer kit (eBioscience 00-5523-00) was used after surface marker staining. Antibodies: cytokeratin 8 (abcam, EP1628Y, 1:1000), cytokeratin 13 (abcam, EPR3671, 1:2000), cytokeratin 14 (ThermoFisherScientific, LL002, 1:100), cytokeratin 15 AlexaFluor555 (abcam, EPR1614Y, 1:500).

### Surface Marker Screening Assay

For the Legend Screen (BioLegend 700001), CD45^+^ cells were partially depleted from digested tissue (15x10^8^ cells) anti-human CD45 beads (Miltenyi 130-045-801) and mixed with 7x10^7^ undepleted cells. Cells were pre-mixed with antibodies against CD45 (BioLegend, 2D1) and EpCAM (BioLegend, 9C4).

### Flow Cytometry Sample Acquisition and Data Processing

All flow cytometry data were acquired on a BD LSR Fortessa (BD Biosciences). Data were analysed using FlowJo (version 10).

### Cell Sorting

For fluorescence-activated cell sorting (FACS), thymic stromal cells were enriched by Percoll-density gradient centrifugation (25) followed by depletion of CD45^+^ cells from the APC-enriched cell suspension (5x10^8^ cells) with anti-human CD45 beads (Miltenyi 130-045-801).subsequently stained with fluorochrome-conjugated antibodies. This stepwise enrichment lead to an approx. 200-fold proportional enrichment of TECs, fibroblasts and endothelial cells (**Figures S2K, L**). Propidium iodide was added to assess cell viability. 1-2x10^5^ live cells were sorted on a FACSAria fusion (BD Biosciences) and resuspended in RLT lysis buffer (Qiagen).

### RNA Extraction and Reverse Transcription to cDNA and qPCR

Ribonucleic acid (RNA) was extracted with the Qiagen RNeasy micro kit (Qiagen, 74004). Reverse transcription was performed with theQiagen QuantiNova Reverse Transcription kit (Qiagen, 205413). qPCR was performed with the Qiagen QuantiNova qPCR master mix (208154) (on a 900HT Fast Real-Time PCR System (Applied Biosystems). Primers were used at a concentration of 10 µM: AIRE forward 5’-CAA GGA TGA CAC TGC CAG TC-3’, reverse 5’-TGC TCT GGA TGG CCC ACT G-3’, reference genes: GAPDH forward 5’-CTT CAA CAG CGA CAC CCA CT-3’, reverse 5’-TGCTGTAGCCAAATTCGTTGTC-3’, HPRT-1 forward 5’-CCT GGC GTC GTG ATT AGT G-3’, reverse 5’-TCG AGC AAG ACG TTC AGT CC-3’, RPLP13 forward 5’-CGG ACC GTG CGA GGT ATG CT-3’, reverse 5’-AGC AGG AAC CAC CAT CCG CT-3’. Results were analysed using the 2^-ddCt^ method (26).

### Library Preparation

The SMARTer^®^ Stranded Total RNA-Seq Kit v2 -Pico Input Mammalian (A Takara Bio Company, California, USA) was used for total RNA samples (0.25–10 ngThe quality and quantity of the enriched libraries were validated using a Qubit^®^ (1.0) Fluorometer and the Tapestation (Agilent, Waldbronn, Germany). The libraries were normalized to 10nM in Tris-Cl 10 mM, pH8.5 with 0.1% Tween 20.

### Sequencing on NovaSeq 6000

After library quantification, libraries were prepared for loading accordingly to the NovaSeq workflow with the NovaSeq 6000 Reagent Kit (Illumina, Catalog No. 20012865).

Cluster generation and sequencing were performed on a NovaSeq 6000 System with a run configuration of single end 100bp.

### Bioinformatic Processing of Transcriptomic Data

The raw reads were first cleaned by removing adapter sequences, trimming low quality ends, and filtering reads with low quality (phred quality <20) using Trimmomatic (Version 0.36) (27). Sequence pseudo alignment of the resulting high-quality reads to the Human reference genome (build GRCh38.p10, Ensembl release 91) and quantification of gene level expression was carried out using Kallisto (Version 0.44) (28).

The normalized signal was internally calculated by EdgeR using the TMM method (29).

Raw data is publicly accessible at the European Nucleotide Archive database under accession number PRJEB39649.

### Statistical Analysis of Transcriptomic Data

For statistical comparison a QL (Quasi-likelihood) F-Test was used to calculate p values. The pValue adjustment was performed with the Benjamini-Hochberg method (30). The log2 Ratio was calculated based on TMM normalised counts using the sample group mean values.

### Immunofluorescence Staining of Human Thymic Tissue

Thymic tissue (fresh or fixed with 4% paraformaldehyde) was embedded in OCT (Biosystems Switzerland, 81-0771-00) and snap-frozen in methanol/dry ice Tissue blocks were cut into 8 μm sections onto Superfrost Plus Slides (Thermo Scientific).

Samples were fixed with ice-cold methanol for 4 minutes (AIRE, cytokeratins) or aceton (collagen IV α5, α6) or 4% paraformaldehyde followed by permeabilization with 0.25% Triton-X100 (CD200, collagen IV α3, FN1 and CD49f). For pdpn, heat-mediated antigen retrieval was performed at pH 6 in 20 mM Citrate buffer for 5 minutes.

All samples were blocked prior to the staining procedure with PBST + 5% fetal calf serum Fluorochrome conjugated antibodies were: cytokeratin 8 AlexaFluor488 (abcam, EP1628Y, 1:100), cytokeratin 5 AlexaFluor647 (abcam, EP1601Y, 1:100), cytokeratin 13 AlexaFluor647 (abcam, EPR3671, 1:100), cytokeratin 14 FITC (invitrogen, LL002, 1:50), cytokeratin 15 AlexaFluor555 (abcam, 1:100). Unconjugated antibodies were: AIRE (abcam, ab78965 goat polyclonal, 1:100), pdpn (abcam, 18H5 mouse monoclonal, 1:100), CD200 (Invitrogen, PA5-47375 goat polyclonal, 1:50), COL4A3 (abcam, ab223227, goat polyclonal, 1:25), CD49f (Biolegend, GoH3, rat monoclonal, 1:25), FN1 (abcam, IST-9, mouse monoclonal, ab6328). UEA-1 (Sigma-Aldrich L9006) was used at 50 μg/ml.

Secondary antibodies: (donkey anti-goat IgG H&L Alexa Fluor555, abcam, ab150134; goat anti-rabbit IgG H&L, Alexa Fluor647, Invitrogen, A21244; goat anti-rat IgG H+L, Alexa Fluor488, Invitrogen, A11006; goat anti-mouse IgG1, AlexaFluor647, Invitrogen, A21240) diluted 1:1000. 4′,6-diamidino-2-phenylindole (DAPI) at 5 μg/ml was added for nuclei and slides were mounted with Prolong Diamond Antifade Mountant (Molecular Probes P36961).

Images were acquired on a Nikon Ti2-E with a Plan Apo λ 20x Ph2 DM (numerical aperture 0.75) and a Plan Fluor 40x Oil DIC H N2 (numerical aperture 1.3) objective with a DS-Q2 camera and the NIS Elements Advanced Research software. Higher resolution images were acquired on a Leica SP8 inverse confocal laser scanning microscope with a 63x glycerin immersion objective [HCX PL APO 37°C CS2 (numerical aperture 1.3)] at room temperature with the Leica LAS X software.

Images were processed in ImageJ2 (31).

### Graphical Illustration With R

Data plots were generated using the ggplot2 package (32) in R (33) in combination with the tidyr package (34). The Venn Diagram was created using the package “VennDiagram” (35), the heatmap was created using the package “heatmap3” (36).

## Results

### Flow Cytometry Surface Antibody Screen Identifies Novel Markers of Human TECs and Subpopulations

**Figure 1:**
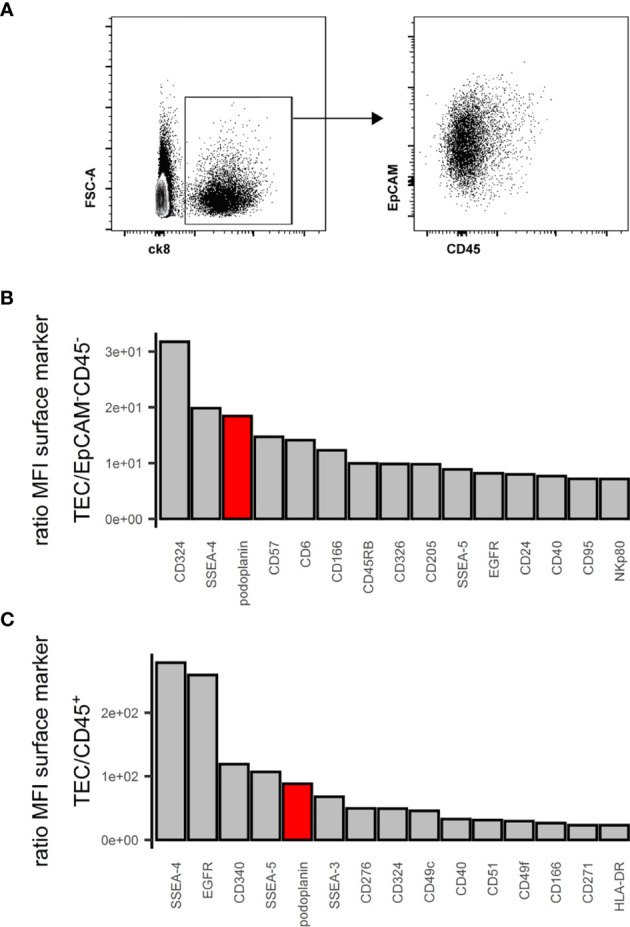
Flow cytometry surface marker screen for comprehensive pan-TEC markers. **(A)** Left plot: Flow cytometry analysis of APC-enriched human thymus cell suspension gated on live single cells. Right plot: ck8^+^ cells. **(B, C)** Top 15 surface markers from surface marker screen based on the ratio of mean fluorescence intensity (MFI) between TEC and EpCAM^-^CD45^-^ cells **(B)** or TEC and CD45^+^ cells **(C)**. pdpn in red.

EpCAM, CD249 and UEA-1 clearly identify murine TECs and their subsets within the CD45^-^ stromal cells (2). In humans, EpCAM expression is variable with cTECs generally expressing lower levels than mTECs (18, 37). Using cytokeratin (ck)8 or ck5 as specific markers of all human TECs (**S1A **and** S1B Figure**) (20, 38), we found that a subset of ck+ cells exhibited very low EpCAM surface levels, especially in thymic cells from some donors (**Figure 1A**), indicating that EpCAM expression alone is insufficient to clearly distinguish human TECs with low-level EpCAM expression from non-TEC stromal cells (**S1C Figure**). Thus, we subjected human thymic stromal cell preparations to an FC surface marker screening (332 markers) aiming at identifying more suitable surface markers for the comprehensive identification of human TEC. Within the CD45^-^ population we assessed the MFI of the interrogated markers, ranked as ratio between the EpCAM^high/int^ vs. EpCAM^-^ population (**Figures 1B, C**). NC-08, an antibody recognizing pdpn, emerged as candidate discriminating TECs from the other thymic cell types in conjunction with EpCAM. The comprehensive coverage of all human TECs using EpCAM and pdpn was confirmed by FC using intracellular staining for ck8 (**S1D Figure**, **S1E Figure**).

**Figure 2:**
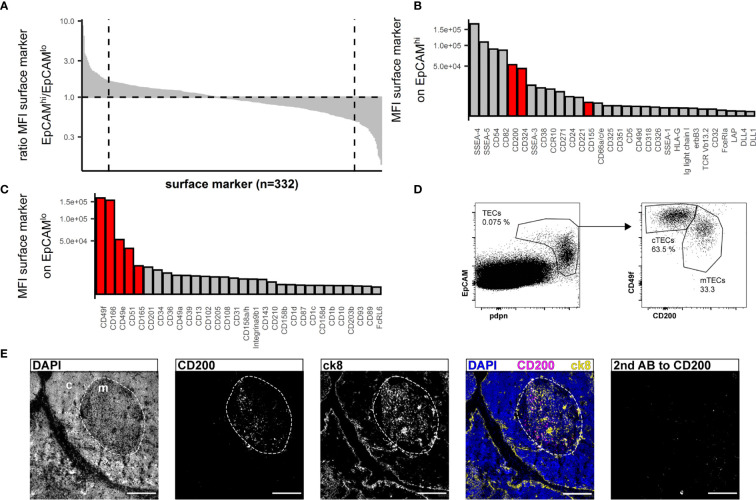
Flow cytometry surface marker screen for markers distinguishing human cTECs and mTECs. **(A)** All screened surface markers plotted based on the ratio of MFI between CD45^-^EpCAM^high^ (putative mTECs) and CD45^-^EpCAM^low^ (putative cTECs). Dashed line: top 30 markers. **(B, C)** Top 30 surface markers from **(A)** with high EpCAM^high^/EpCAM^low^ MFI ratio **(B)** or low EpCAM^high^/EpCAM^low^ ratio **(C)** plotted by absolute MFI. Candidates that were chosen for further evaluation are marked in red. **(D)** Flow cytometry identification of TECs based on EpCAM and pdpn (left plot) and delineation of cTECs and mTECs based on CD49f and CD200 from live single cells from APC-enriched human thymus cell suspension. **(E)** Cryosections of human thymus. Nuclei are visualized with DAPI (blue). Cortex (c) and medulla (m) are distinguished based on density of nuclei (dashed line). Staining for CD200 (magenta). TECs are visualized with antibodies against ck8 (yellow). Exposure time for CD200 was adapted according to the staining with secondary antibody to CD200 (left panel). Scale bar 100 μm.

Human cTECs are commonly identified using antibodies against CD205 (2) or the CDR2-antibody, recognizing a yet unknown epitope (39). UEA-1 was reported to label human mTEC (20). We found that in multiple human thymus samples, the combination of UEA-1 and CD205 left a substantial fraction of TECs unstained (**S1G Figure**), and while UEA-1 staining was exclusively found in the medulla, it failed to label all mTECs (**S1F Figure**, **S1G Figure**). EpCAM expression is reportedly higher on human mTECs (EpCAM^high^) than cTECs (EpCAM^low^) (18, 37). Aware of the caveat that we may omit some EpCAM^low^ cTECs in our analysis, we used the data from the above FC screening assay and interrogated EpCAM^high^ vs EpCAM^low^ TECs (**S2A Figure**) for more distinct yet comprehensive markers. Ranking the ratio of the MFI assessed in EpCAM^high^ vs EpCAM^low^ cells identified markers with high discriminatory capacity between the two populations (**Figure 2A**). The top 30 markers from each end of this spectrum were further evaluated for their absolute MFI, as high expression levels are potentially more suitable for FC (**Figures 2B, C**). Potential markers were confirmed by visual inspection of the FC data based on uniformity of the subpopulations and clear-cut discrimination between them, which led us to exclude CD54 and CD82 (**S2B Figure**). SSEA-4 and SSEA-5 were dismissed as these are glycolipids rather than surface proteins and thus not easily correlated with the expression of particular genes. An additional set of 4 thymi was used to evaluate the best candidates (CD200, CD342, CD155 for mTECs, CD49f, CD166, CD49e, CD51, CD165 for cTECs) for inter-individual reproducibility (**S2C Figure**). Based on these criteria, antibodies reactive to CD200 (on mTEC/EpCAM^high^) and CD49f (on cTEC/EpCAM^low^) respectively proved to be most effective due to high expression levels and low inter-individual variability (**Figure 2D** and **S2C Figure**).

Immunofluorescence analysis of thymus cryosections revealed exclusive medullary staining for CD200 (**Figure 2E**), but not restricted to mTECs. In fact, FB, EC and a small fraction of B cells express CD200 as assessed by FC (**S2E Figure**). The corresponding receptor, CD200R, was mainly found on B cells, macrophages and dendritic cells (**S2F Figure**). Prominent reactivity to CD49f was detected on intrathymic vascular structures by immunofluorescence analysis (**S2D Figure**), rendering it impractical as a discriminatory marker for cTECs in IF analysis. This is in accordance with FC data (**S2J Figure**). However, vascular endothelial cells are easily discriminated from pdpn^+^ cTECs based on pdpn and EpCAM expression (**S2K Figure**). Lymphatic endothelial cells co-express high levels of CD49f and CD200 as well as CD31 and pdpn, allowing their clear distinction from TECs (**S2I, J Figure**).

We thus propose the following strategy for the identification of TEC: pdpn^high/int^EpCAM^high/int/low^ for TECs, with further subdivision of CD49f^+^CD200^-^ for cTECs and CD49f^int/low^CD200^+^ for mTECs. Interestingly, CD45 proved to be dispensable for our gating strategy (**S1D Figure** and data not shown).

Gene expression analysis of *AIRE* and *FOXN1* within accordingly FC-sorted TEC subpopulations and a CD45^-^EpCAM^-^ control cell population demonstrated exclusive expression of *AIRE* in the mTEC subset, whereas *FOXN1* expression was found in both subsets but not in the control cells (**S2G Figure**, **S2H Figure**).

### Transcriptomic Analysis of Bulk Sorted Thymic Stromal Subsets Validates Gating Strategy

**Figure 3:**
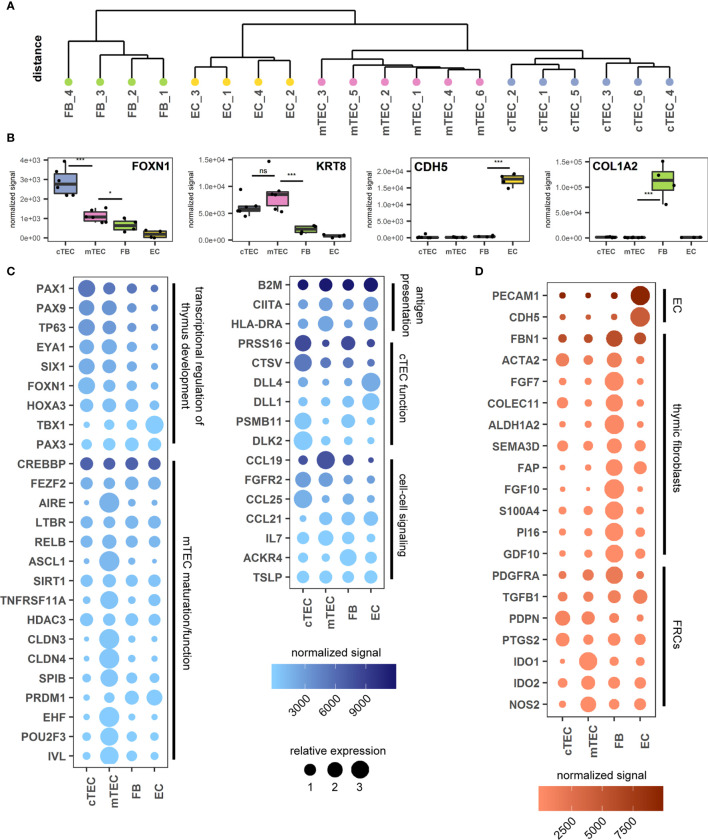
Transcriptomic analysis of thymus stromal populations according to the proposed gating strategy. **(A)** Unsupervised clustering of all gene expression values. **(B)** Genes representative of each sorted cell population. ***p < 0.001, *p < 0.05, ns: p > 0.05. ns, not significant. **(C, D)** Genes relevant for TEC biology **(C)** or FB and EC biology **(D)** according to the sorted cell population. Color gradient reflects the mean expression signal for each gene across all cell populations, dot size represents the relative expression value of each gene between cell populations. Genes are grouped in categories as labelled.

In view of a more profound validation of the proposed gating strategy we sought to gain a detailed understanding of the transcriptional activity of the individual stromal cell subpopulations using RNA-sequencing of bulk sorted subpopulations. To this end we sorted cTECs and mTECs according to our proposed gating strategy (6 individuals) as well as CD45^-^CD31^+^pdpn^-^ vascular endothelial cells (EC) and CD45^-^HLA-DR^-^pdpn^int^ fibroblasts (FB) (4 individuals). Identically sorted cell populations grouped together when unsupervised clustering of normalized gene expression data was applied. Additionally, TEC subpopulations distinctly separated from non-epithelial cell subsets (**Figure 3A**).

The identity of each subset was assessed interrogating characteristic gene expression profiles. In accordance with published data *FOXN1* (cTEC > mTEC) and *KRT8* (ck8) expression was increased in TECs, whereas *CDH5* (VE-Cadherin) and *COL1A2* were found exclusively in ECs and FBs respectively, thus confirming the identity of the subpopulations sorted according to the novel gating strategy (**Figure 3B**) (16, 20). Gene expression levels of the markers used for FC identification and sorting, *EPCAM* (EpCAM), *PDPN* (pdpn), *ITGA6* (CD49f) and *CD200* (CD200) were distributed according to the expected pattern (**S3A Figure**).

Furthermore, markers associated with thymocytes, *CD7* (CD7) and *PTPRC* (CD45) (16), were only detectable at very low levels in all four cell populations (**S3B Figure**), suggesting minimal contamination thereof. According to recent data from scRNA-sequencing there is substantial heterogeneity with both cortical and medullary TECs (16, 18, 40). We therefore assessed our dataset from bulk-sorted cTEC- and mTEC subpopulations for signatures associated with proposed subsets. The distribution of genes associated with tuft-like cells (POU2F3, IL25), neuroendocrine cells (NEUROD1, CHGA), myoid cells (MYOG, MYOD1, BEX1), corneocyte-like cells (FXYD3, LYPD2, IL1RN), ciliated cells (SOX2, FOXJ1), immature TECs/cTECs (CTGF, ZBED2) or mTECs (ASCL1, INSM1) were found to be upregulated in the respective TEC population (**S3C Figure**).

Medullary TECs contribute to self-tolerance through the expression of TRAs at very low levels (2, 37). Furthermore, each TRA is only expressed in about 1-3% of all mTECs (37). We therefore looked for low-level unique gene expression across all samples. All genes expressed at >0.5 fragments per kilobase of transcript per million mapped reads (FPKM) in at least 3 out of 6 (cTEC and mTEC) or 2 out of 4 (FB and EC) samples were checked for their expression across all four cell populations. Housekeeping genes -as defined by the human protein atlas- were excluded (**S3D Figure**). The mTEC subset demonstrated by far the highest number of exclusive transcripts (841), compared to 128 in cTECs, 227 in FB and 104 in EC. Comparing these 841 genes to the human protein atlas tissue specific gene database most of them (796) could be assigned to one of the tissue-specificity categories (**S3E Figure**). The 263 genes that fell into the “tissue enriched” category contained well-known TRAs such as Insulin (not shown). The highest number of genes could be assigned to brain (41), followed by testis (42). Other organs with a high number of TRAs (liver, kidney, pancreas) were also represented (**S3F Figure**), confirming a hallmark feature of this population sorted according to the proposed strategy.

Thymic stromal cell development and differentiation is guided by a number of transcriptional regulators, which control the expression of factors involved in cell-type specific growth and function (**S1 Table**). We therefore analyzed the expression levels of genes with known and suspected involvement in transcriptional regulation of thymus development, mTEC maturation/function, antigen presentation, cTEC function, cell-cell signaling from mouse or human studies (**Figure 3C** and **S1 Table**).

In line with previous findings the expression of transcription factors and regulators associated with thymic development were up-regulated in TEC subpopulations with the exception of *TBX1* and *PAX3*, which are involved in patterning of the pharyngeal pouches. Whereas *AIRE*, *ASCL1*, *EHF*, *POU2F3* and *SPIB* expression were restricted to mTEC, neither the transcription factor *FEZF2* nor the transcriptional regulators *SIRT1* or *PRDM1* demonstrated specific up-regulation in this bulk-sorted population. In accordance with published observations (20, 43), expression of the mTEC-associated tight junction proteins *CLDN3* and *CLDN4* was restricted to the mTEC population.

The analysis related to phenotypic and functional characteristics of fibroblast and endothelial cells demonstrated high levels of *PECAM1* (CD31) and *CDH5* (VE-cadherin) expression in endothelial cells as expected (16). The CD45^-^EpCAM^-^HLA-DR^-^pdpn^int^ population uniquely expressed fibroblast-associated markers (including *FAP*, *S100A4*, *PDGFRA*) and contains the markers described for the populations Fb1 (intrathymic FB) (*COLEC11*, *GDF10*, *ALDH1A2*) and Fb2 (capsular FB) (*FBN1*, *PI16*, *SEMA3D*) (18). Notably, the fibroblast population distinctively expressed high levels of *FGF7* and *FGF10*, known for their contribution to TEC proliferation and differentiation (9) (**Figure 3D**), whereas the corresponding receptor (*FGFR2*) is highly upregulated in both cTECs and mTECs (**Figure 3C**). Interestingly, RNA levels of molecules involved in immunomodulatory function of fibroblastic reticular cells (FRCs) in the periphery (such as *PTGS2*, *IDO1* and *IDO2* and *NOS2* (44) are more pronounced in TECs.

In murine studies, co-stimulatory molecules (such as CD80) are commonly used in conjunction with MHCII expression to identify mTEC maturation stages (42). However, on human TECs we were unable to detect protein expression of CD80, CD86 or OX40L in our FC surface marker screen (**S3G Figure**). Another costimulatory molecule, CD40, was detected at high levels on both cTECs and mTECs (**S3G Figure**), although literature suggests an exclusive expression on human mTECs (45). Tissue preparation may affect epitope availability, although expression of each of these molecules was detectable on CD45^+^ cells (data not shown). In accordance with our surface marker screen, transcript levels of CD80, CD86, and OX40L were very low. Yet, mRNA and protein levels for CD40 were found to a similar extent on both TEC subsets (**S3G Figure**).

### TEC-Mediated Sub-Compartmentalization of the Thymus

Cytokeratins (cks) are critical for epithelial cell structure and function. Our dataset showed robust differential expression for ck13 (**S4A Figure**), ck14 (**S4B Figure**) and ck15 (**S4C Figure**). The restriction of ck13 expression to mTECs was confirmed by FC and IF, yet found to be independent of AIRE expression. On the other hand, ck14 and ck15 were found in almost all TECs by FC and IF (**S4 Figure** and data not shown).

**Figure 4:**
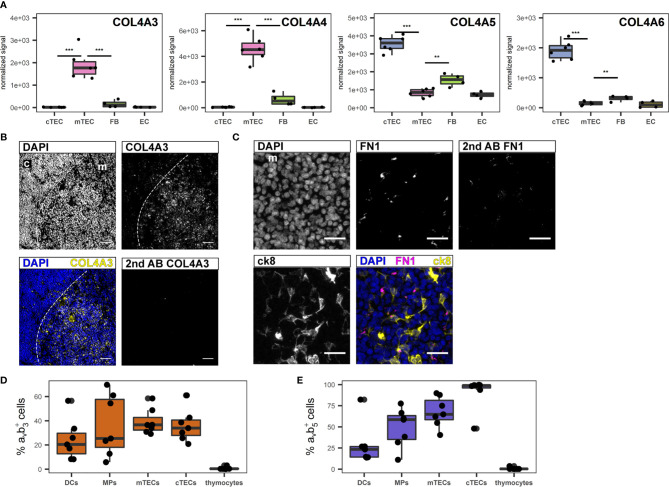
Extracellular matrix genes differentially expressed between human cTECs and mTECs. **(A)** Gene expression data for *COL4A3*, *COL4A4*, *COL4A5* and *COL4A6*. ***p < 0.01, **p < 0.01. **(B, C)** Cryosections of human thymus. Nuclei are visualized with DAPI (blue). Cortex (c) and medulla (m) are distinguished based on density of nuclei (dashed line). Exposure time was adapted according to staining with secondary antibody. **(B)** Staining for COL4A3 (yellow). Scale bar 100 μm. **(C)** Staining for FN1 (magenta) in medullary area (m). TECs are visualized with ck8 (yellow). Scale bar 20 μm. **(D, E)** Percentage of positive cells for integrin α_v_β_3_ **(D)** and α_v_β_5_ **(E)** of indicated cell populations determined by flow cytometry from human thymus cell suspension. mTECs and cTECs were gated as described. Dendritic cells (DCs) were gated as CD45^+^HLA-DR^+^CD11c^+^CD11b^+^, macrophages (MPs) were gated as CD45^+^HLA-DR^+^CD11c^-^CD11b^+^. Thymocytes were gated as CD3^+^ and/or CD1a^+^.

For their growth and migration developing thymocytes rely on specialized and highly organized ECM produced by stromal cells (46). Even though the three-dimensional network structure of TECs is devoid of a classical basement membrane, collagen IV, fibronectin and laminin, which are the principal components of basement membranes are reportedly part of the thymic ECM (47, 48). The most significant contribution to gene transcripts associated with the GO term “extracellular matrix” came from FB and EC, and included collagen IV subtypes COL4A1 and A2 (**S5A Figure**, **S5B Figure**). Interestingly, the α3 to α6 chains of collagen IV ranked among the most differentially regulated genes in cTECs versus mTECs (**Figure 4A**). Thus, while the contribution of TECs to this gene set seems to be limited, it is highly subset-specific, with COL4A3 and COL4A4 expression being significantly higher in mTECs, whereas COL4A5 and COL4A6 are expressed predominantly in cTECs (**Figure 4A**). Corresponding protein expression was confirmed by IF (**Figure 4B** and **S5C Figure**, **S5D Figure**). Gene expression of FN1 (fibronectin) was most prominently expressed in TECs in comparison to other stromal cells (**S5A Figure**, **S5B Figure**), yet detection of the protein was found mainly in the medulla (**Figure 4C**) in close proximity to mTECs (**S5E Figure**). Collagen IV fibres of the basal membrane serve as structural framework for epithelial cells and other cell types with distinct integrins linking the cytoskeleton to specific collagen heterotrimers (49). In particular, α_v_β_3_ and α_v_β_5_ integrins bind to the α3α4α5 or the α5α5α6 heterotrimer of collagen IV (49). As the prevalence of different collagen IV heterotrimers seemed to be specific for either thymic cortex or medulla, we investigated the presence of both integrin dimers on several thymic cell types. Whereas thymocytes were devoid of either integrin expression, a large fraction of stromal cells including dendritic cells (DCs), macrophages (MPs) and TEC subsets expressed one or both integrins (**Figures 4D, E**).

### Sex-Specific Alterations of TEC and Thymocyte Populations in Infants

Thymic pathology can be observed in a number of inborn or acquired conditions such as microdeletion 22q11.2 syndrome, myasthenia gravis (MG), or high-dose steroid treatment (50–52). We therefore assessed the applicability of our TEC identification strategy on thymic biopsies from patients with these entities. The established gating strategy reliably identified TECs and their subpopulations in these patients. It also revealed significant alterations of MHC class II expression in both conditions compared to an immunologically healthy control, with markedly lower (MG patient) or very low (22q11.2 microdeletion patient) HLA-DR expression on both cTECs and mTECs (**S6A Figure**, **S6C Figure**, **S6E Figure**). The cytokeratin distribution in the thymus biopsy from the patient with MG was severely altered, with disruption of the epithelial network and aberrant cortico-medullary demarcation compared to a healthy control (**S6B Figure**, **S6D Figure**). The thymus from the patient with microdeletion 22q11.2 on the other hand demonstrated normal thymic architecture and inconspicuous distribution of cytokeratins (**S6F Figure**).

**Figure 5:**
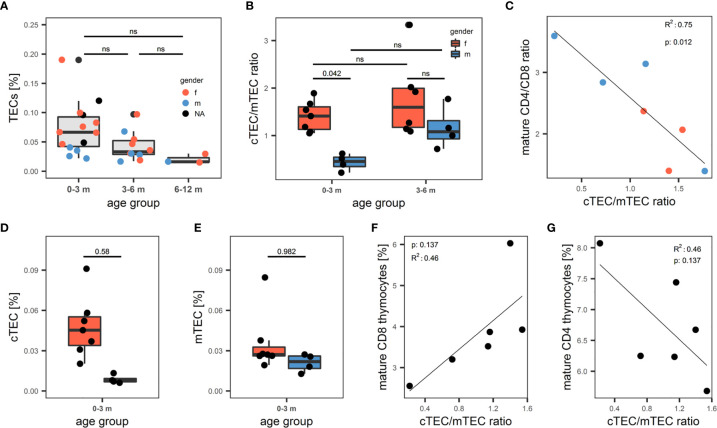
Quantification of human thymic stromal cell populations by flow cytometry. **(A-G)** Flow cytometry quantification of thymic cell populations. TECs were gated as described above from non-enriched cell suspensions. **(A)** Percentage of TECs in human thymus cell suspension across different age groups. N = 31. Dots are coloured based on sex of the donor. Red: female. Blue: male. NA: information on sex not available. **(B)** cTEC/mTEC of thymi from **(A)** across different age groups based on gender. Red: female. Blue: male. **(C)** Correlation between cTEC/mTEC ratio and the ratio between mature CD4^+^/CD8^+^ thymocytes. Mature thymocytes were gated as CD3^high^CD1a^int/low^. Color of dots represents gender of donor as in **(A)**. R^2^ and p value derived from R^2^ analysis are depicted top left. **(D, E)** Absolute percentage of cTECs **(D)** and mTECs **(E)** in the 0-3 months age group. Color of the box represents gender of the donor as in **(B)**. **(F, G)** Correlation between cTEC/mTEC ratio and the percentage of mature CD8^+^ **(F)** or CD4+ **(G)** thymocytes of total thymocytes. Thymocytes were gated on live single cells as CD3^+^ or CD1a^+^, mature thymocytes were gated as CD3^high^CD1a^int/low^. ns, statistically not significant.

Having established a reliable and comprehensive identification strategy for human TECs enabled us to quantify TECs by FC in a total of 31 thymi from donors age 1 day to 12 months. The median fraction of TECs in human thymus decreased – though not reaching statistical significance - from 0.067% in the 0-3 months old infants, to 0.033% in patients aged 3-6 months, to 0.016% in the 6-12 months age group (**Figure 5A**). Dendritic cell frequency (CD45^+^HLA-DR^+^CD11c^+^CD11b^+^) and LEC (CD45-CD31+pdpn+) followed the same trend, whereas the proportion of macrophages (CD45^+^HLA-DR^+^CD11c^-^CD11b^+^), EC (CD45-CD31+pdpn-) remained constant in these age groups (S7A-E). Averaged across the age groups, we found median frequencies of 0.044% (MP), 0.057% (DC), 0.06% (FB), 0.061% (EC) and 0.0013% (LEC) in native thymi. Interestingly, male infants (age 0-3 months) demonstrated a significantly decreased cTEC/mTEC ratio due to a disproportionate decrease of the cTECs in comparison to mTEC frequency in this group of patients (**Figures 5B–E**). Thymocyte development and TECs are connected in a mutual regulatory network, a.k.a. thymic crosstalk (11). Hence, we sought to identify alterations in the thymocyte compartment associated with the observed changes in the TEC compartment. We found a significant inverse correlation when comparing the ratio of mature (CD3^high^CD1a^low^) CD4- to CD8-single-positive thymocytes with the cTEC/mTEC ratio (**Figure 5C**). The frequency of mature (CD3^high^CD1a^low^) CD8-single-positive thymocytes showed a trend for increase with the surge of the cTEC/mTEC ratio, whereas the opposite was seen for the mature CD4-single-positive counterpart (**Figures 5F, G**).

## Discussion

### EpCAM, pdpn, CD200 and CD49f Reliably Identify Human TECs by Flow Cytometry

In the present study we identified surface markers useful in the comprehensive flow-cytometric identification of human TECs (pdpn in conjunction with EpCAM) as well as their reliable partition into cortical and medullary subpopulations (CD49f and CD200). The combination of these markers provided unambiguous positive identification of all TECs, even those with low EpCAM expression, and demonstrated high inter-individual reproducibility.

Previous strategies to analyse human thymic tissue were mostly based on histological analysis (20, 23), or on thymocyte and peripheral T cell populations subpopulations as indirect markers (53). By contrast, FC provides a simple and relatively fast method for tissue analysis. We thus took advantage of our approach to analyse 31 thymi from children in their first year of life. We found a pronounced reduction in cTEC/mTEC ratio in males younger than 3 months of age compared to age-matched females, which resulted from a relative lack of cTECs rather than an increased proportion of mTECs. Androgens are known to negatively impact on TEC proliferation and regeneration (52, 54), and especially impair cTEC regeneration (54, 55), whereas estrogens have a negative effect on mTECs (56).

In humans, minipuberty, a physiological post-natal surge in androgens in male infants younger than 6 months (57), has been correlated with sex differences in thymus function in human infants (58, 59). Thus, our findings corroborate these reports on the effects of gonadal steroids on TEC composition, suggesting a negative impact of androgens on the frequency of cTECs, which might explain previously observed relative increases in mTECs and AIRE expression in human male infants.

Peripheral T cell function is crucial for efficient immune response and prevention of autoimmunity. As these might be directly related to thymic output and thymocyte development (60), we were interested whether the androgen-related change in thymic stroma composition could have an influence on thymocyte populations. We found that the cTEC/mTEC ratio was negatively correlated to the ratio between mature CD4 and CD8 SP thymocytes. As CD8+ thymocytes require longer cortical dwell time, these cells might benefit from a larger thymic cortex (61). Studies in mice demonstrated that different proteolytic enzymes are responsible for the generation of either CD4 and CD8 thymocytes, highlighting the importance of cTECs for thymocyte development (62, 63). Along these lines, patients with thymoma of preferential cortical phenotype demonstrate an increased proportion of CD8+ T cells in the peripheral blood (64). On the other hand, androgens may have a direct effect on thymocyte development (65).

Our proposed approach to comprehensively quantify human TEC subpopulations by FC may contribute to a better understanding of stromal changes in diseases associated with thymic defects, as demonstrated in samples derived from patients with microdeletion 22q11.2 and myasthenia gravis (50, 51). Also, it may easily be applied to the evaluation of thymic grafts pre- and post-transplantation and thus complement currently used histological methods (24).

### Biological Significance of CD200, CD49f and pdpn in Human Thymus Biology

The thymic expression pattern of CD200 with its restriction to the medulla and its high expression on all mTECs raises several questions as to its potential contribution to human T-cell development. In mice, this differential expression pattern was not observed (data not shown). CD200 was initially discovered as a T-cell co-stimulatory molecule, but was later shown to induce an immunomodulatory program in antigen-presenting cells and is thus considered an immune checkpoint molecule (41). CD200 expression on various hematological and solid tumors may contribute to immune evasion partly due to induction of regulatory T cells (66–68). Although a direct effect of CD200 expressed by mTECs on thymocytes is unlikely – the corresponding receptor is lacking on thymocytes (data not shown) – it may induce a modulatory effect on thymic medullary APCs, some of which express the corresponding receptor (data not shown).

The integrin CD49f has previously been described as a marker for human TECs (69, 70). We found that addition of CD49f to our flow cytometry approach proved valuable for a better discrimination between cTECs and mTECs, as the latter generally show a lower CD49f expression level.

Taking into account the expression of pdpn restricted to an mTEC precursor population at the cortico-medullary junction in mice (71), the generic distribution on human TECs was unanticipated, but suggests significant species-specific differences in its regulation of expression and potential biological function. This evolutionarily conserved glycoprotein is critical for the development of the lymphatic system and lymphoid organs and has been detected in healthy and diseased human thymus previously (72). It contributes to cell adhesion and interacts with soluble and cell-bound proteins (including chemokines) and contributes to the local accumulation and formation of gradients of growth and differentiation factors (73). In murine lymph nodes, pdpn orchestrates the migration of DCs and tissue homeostasis directly (through interaction with CLEC2) and indirectly (through gradient formation) (74). By sequestration of the CCR7 ligand CCL21 on murine thymic fibroblasts, pdpn is involved in the formation of regulatory T cells (75). The broad function of pdpn in lymphatic organs suggests that it also plays a vital role in human TEC biology, either by interaction with growth factors or by distribution and function of DCs.

In accordance with numerous studies in mice and humans, transcripts associated with central tolerance (AIRE and TRAs) were upregulated in mTECs. However, we found significant differences to observations derived from murine studies. Neither *FEZF2*, *SIRT1* and *HDAC3* were differentially expressed by specific stromal cell subpopulations (see **S1 Table** for references). FEZF2 was shown to regulate AIRE-independent clusters of TRAs, and both molecules were important for self-tolerance in mice (76). In comparison to other human thymic APC, expression was reported exclusively in mTECs (76, 77). In our study with a focus on non-HSC-derived stromal cells we find that *FEZF2* is similarly expressed in all four stromal cell populations, however this might also be due to our bulk sorting and analysis strategy. Taken together, we have identified several novel molecules on human thymic stromal cells that differ from their expression in the mouse counterparts.

### TEC Function Beyond Positive and Negative Selection

Murine mTEC maturation is in parts defined by the expression of co-stimulatory molecules such as CD80, CD86, CD40 and OX40L, which are critical for thymic crosstalk and induction of thymic tolerance in mice. Especially CD80 is often used in conjunction with MHC II to determine the stage of mTEC maturation, but for human thymus, this has not been described so far (11, 42). Neither gene expression nor surface protein expression analysis revealed substantial levels of these molecules on mTECs in our dataset, with the notable exception of CD40, which we also found on FB and EC, although initial evidence from human thymus suggested CD40 expression restricted to mTECs (45). Previous reports of costimulatory molecules in the human thymus have highlighted their expression by non-epithelial APCs and their importance for Treg generation (78–80), indicating that their expression on mTECs might be dispensable and thus has limited suitability to serve as maturation markers in the human organ as opposed to mouse thymus.

Both major TEC subsets exert specialized functions with vastly differing molecular mechanisms. The functional dichotomy in the thymus is underscored by the histological partition into distinct compartments. In polarized epithelia, collagen IV is the hallmark collagen of the basal membrane (49), but the thymus is devoid of a clearly polarized architecture (81). The presence of the collagen IV α3 chain has been described before in human thymus Hassall’s corpuscules, but was considered a TRA (82). Furthermore, collagen IV was detected in the human thymus, but mainly localized to the septum and blood vessels (47). This might be due to strong expression of *COL4A1* and *COL4A2* by fibroblasts and endothelial cells according to our data. Even though our transcriptomic analysis revealed that in the thymus the bulk of ECM is contributed by fibroblasts and endothelial cells, we found that differing production of collagen IV α-chains and fibronectin by cTECs and mTECs might aid to shape the compartmentalization into thymic microenvironments. Fibronectin expression has been shown in the thymic medulla and as an important factor for thymocyte-TEC adhesion *in vitro* (47, 83), although the cellular source of fibronectin in the thymus had not been investigated.

Our data suggest that TECs produce a restricted set of ECM components in a highly compartment-specific fashion. Those might serve as adhesion cues for developing thymocytes, which require different adhesion structures during different phases of their development (84). In addition, thymic stromal cells themselves are likely to rely on these structures, as demonstrated here by their expression of integrins specific for collagen IV trimers (49). Whether this interaction provides mere adhesion or is also involved in the commitment and/or maintenance of TEC subsets remains to be clarified. Understanding the role of compartment-specific ECM in thymic function may have important implications in the production of matrices for organoids (46, 85) or in the use of decellularized thymi for the generation of thymic grafts (70).

In lymphatic organs of mice, which are devoid of epithelial cells, FRCs and lymphatic endothelial cells express the highest levels of pdpn (44). Intrathymically, we found the highest levels of pdpn on lymphatic endothelial cells followed by TECs, which prompted us to further investigate overlapping features between fibroblasts and TECs. We found genes characteristic for FRCs (*IDO1, IDO2, NOS2*) in TECs, and genes characteristic for TEC (*IL7, CCL21*) in FB. Recent analysis of cultured human TECs also indicated a partly shared gene expression signature between TECs and mesenchymal cells (70). The picture emerging from these studies (reviewed in (6) and our results demonstrate that non-hematopoietic stromal cells (TECs, FB and EC) participate in a complex division of labor with partly overlapping functions similar to what has been described for secondary lymphoid organs (44).

Future studies aimed at investigating redundant and distinctive roles of human thymic stromal cells will aid to define strategies for thymic regeneration and improved graft preparation. Providing detailed phenotypic and transcriptional characterization of the key stromal cell subsets of the human thymus – with a particular focus on TECs - our study significantly contributes to this endeavor.

## Data Availability Statement

The datasets presented in this study can be found in online repositories. The names of the repository/repositories and accession number(s) can be found below: https://www.ebi.ac.uk/ena, PRJEB39649.

## Ethics Statement

The studies involving human participants were reviewed and approved by Ethical Commission Zurich, No 2017-00312. Written informed consent to participate in this study was provided by the participants’ legal guardian/next of kin where applicable.

## Author Contributions

VH performed the majority of experiments, designed the study and wrote the manuscript. MM performed the library preparation and sequencing. LO performed the bioinformatic data processing and helped with the subsequent analysis of the data. HD provided essential tissue samples. SV contributed to the interpretation of data. MH-H designed the study and wrote the manuscript. All authors contributed to the article and approved the submitted version.

## Funding

Our research was funded in parts by the Prof. Max Cloëtta Foundation (MH-H), the Swiss National Science Foundation (VH, #310030_162602) and the Starr Foundation.

## Conflict of Interest

The authors declare that the research was conducted in the absence of any commercial or financial relationships that could be construed as a potential conflict of interest.

## Publisher’s Note

All claims expressed in this article are solely those of the authors and do not necessarily represent those of their affiliated organizations, or those of the publisher, the editors and the reviewers. Any product that may be evaluated in this article, or claim that may be made by its manufacturer, is not guaranteed or endorsed by the publisher.

## Supplementary Material

Supplementary Figure 1 Thymic epithelial cells can be defined by expression of ck8 and ck5, but not distinguished based on expression of CD205 or binding to UEA-1. **(A, B, F)** Cryosections of human thymus. Nuclei are visualized with DAPI (blue). Cortex (c) and medulla (m) are distinguished based on density of nuclei. Asterisks: Hassal’s corpuscules. **(A)** TECs are visualized with antibodies against pan-ck (magenta) and ck8 (yellow). Scale bar: 200 μm. **(B)** TECs are visualized with antibodies against ck5 (magenta) and ck8 (yellow). Scale bar: 200 μm. **(C-E, G)** Flow cytometry staining of APC-enriched human thymus cell suspension gated on live single cells. **(C)** Staining for EpCAM and CD45 **(D)** Left plot: Live single cells stained as shown in **(C)** Right plot: EpCAM and pdpn fluorescence signal of ck8+ cells. **(E)** Right plot: ck8 fluorescence signal of EpCAMhigh/int/lowpdpnhigh/int cells. **(F)** Flow cytometry staining of APC-enriched human thymus cell suspension for CD205 and UEA-1 gated on live single TECs (defined as EpCAMhigh/int/lowpdpnhigh/int). **(G)** Staining with UEA-1 (magenta). TECs are visualized with antibodies against ck5 (yellow). Scale bar: 100 μm.

Supplementary Figure 2 Supplementary data on surface marker screen and cell sorting. **(A)** Left plot: Surface marker screen flow cytometry gating strategy to define TECs (CD45-EpCAM+), FB (CD45-EpCAM-) and CD45+ (CD45+) cells. Gated on single cells from partially CD45+ depleted cell suspension from human thymus. Right plot: Delineation of EpCAMhigh and EpCAMlow cells gated on TECs. **(B)** Individual flow cytometry plots of markers of interest from surface marker screen. Gated on TECs from **(A)**. **(C)** Markers chosen for further evaluation and stained on four additional consecutive thymi. MFIs on CD45-EpCAMhigh and CD45-EpCAMlow cells are compared by paired t-test. **(D)** Cryosections of human thymus. Nuclei are visualized with DAPI (blue). Cortex (c) and medulla (m) are distinguished based on density of nuclei (dashed line). Staining for CD49f (magenta). TECs are visualized with antibodies against ck8 (yellow). Exposure time for CD49f was adapted according to the staining with secondary antibody to CD49f (left panel). Scale bar 50 μm. **(E, F)** Mean fluorescence intensity (MFI) of CD200 **(E)** and CD200R **(F)** on thymic cell populations. Gated on live single cells. MP, macrophages; DC, dendritic cells. **(G, H)** Relative mRNA expression of cell-sorted putative cTECs, mTECs and CD45-EpCAM- cells. Fold change is calculated relative to the median expression level of all samples. **(G)** AIRE mRNA expression. **(H)** FOXN1 mRNA expression. **(I-K)** Fluorescence signal (flow cytometry) of TECs (EpCAMhigh/int/lowpdpnhigh/int, blue), endothelial cells (CD31+pdpn-, green) and lymphatic endothelial cells (CD31+pdpn+, orange) for CD31 (I), CD49f (J) or EpCAM and pdpn **(K)**. **(L, M)** Frequency of TEC, FB and EC (% of live cells) in cell suspensions (digest) prior to (L) and after percoll-density- and CD45-bead-based enrichment **(M)**.

Supplementary Figure 3 Validation of the flow cytometry gating strategy and expression of costimulatory molecules. **(A, B)** boxplot representation of genes from the transcriptomic dataset. ***p < 0.001, **p < 0.01, *p < 0.05, ns, not significant. **(A)** Genes coding for markers used in the sorting strategy. **(B)** Genes characteristic for thymocytes or hematopoietic cells. **(C)** Genes associated with TEC subpopulations according to the sorted cell population. Colour gradient reflects the mean expression signal for each gene across all cell populations, dot size represents the relative expression value of each gene between cell populations. Genes are grouped in categories as labelled. **(D)** Genes that are expressed in at least 3 out of 6 (mTEC and cTEC populations) or 2 out of 4 (FB and EC populations) at FPKM >0.5. **(E)** Genes from **(D)** exclusively expressed in mTECs compared to the human protein atlas tissue specificity categories. The number of matched genes for each category is given in brackets. **(F)** Genes from the tissue enriched category in **(E)** are plotted according to their organ. Number of matching genes for each organ is given in brackets. **(G)** Result for co-stimulatory molecules in transcriptomic dataset (left column) and CD45- cells (gated on single cells) from the flow cytometry surface marker screen (right column). Boxplots: ***p < 0.001, *p < 0.05, ns, p > 0.05. ns, not significant.

Supplementary Figure 4 Cytokeratins as markers for human TEC subsets. **(A-C)** left panel: Gene expression data from the transcriptomic dataset for KRT13 **(A)**, KRT14 **(B)** and KRT15 **(C)**. ***p < 0.001. **(A-C)** right panel: flow cytometry analysis of cytokeratin 13 (ck13), 14 (ck14) and 15 (ck15) on 5 thymi (ck13, ck14) or 3 thymi (ck15). cTECs and mTECs were gated according to the proposed gating strategy. **(D-F)** Cryosections of human thymus. Nuclei are visualized with DAPI (blue). Cortex (c) and medulla (m) are distinguished based on density of nuclei (dashed line). Perivascular space (pvs) indicated by dotted line. **(D)** Staining for ck13 (magenta). TECs are visualized with ck8 (yellow). Scale bar 200 μm. **(E)** High resolution image of thymic medulla. Staining for ck13 (magenta), staining for AIRE (cyan). TECs are visualized with ck8 (yellow). Scale bar 20 μm. **(F)** Staining for ck13 (magenta), ck14 (yellow) and ck15 (cyan). Scale bar 100 μm. Asterisk: ck13+ck14+ck15+ cell. Arrowhead: ck14+ cell. Circle: ck15+ cell.

Supplementary Figure 5 Supplementary information for gene expression profile of ECM components. **(A)** Heat map of the 30 genes with highest variance between all four cell populations that are associated with the GO term “extracellular matrix”. **(B)** Gene expression data for COL4A1, COL4A2 and FN1. ***p< 0.01, ns: not significant. **(C-E)** Cryosections of human thymus. Nuclei in blue. Cortex (c) and medulla (m) are distinguished based on density of nuclei (dashed line). Exposure time was adapted according to staining with secondary antibody. **(C)** Staining for Collagen IV α5 (yellow). Scale bar 200 μm. **(D)** Staining for Collagen IV α6 (yellow). Scale bar 200 μm. **(E)** Staining for FN1 (magenta). TECs are visualized with ck8 (yellow). **(E)** Scale bar 200 μm.

Supplementary Figure 6 Analysis of thymus tissue from patients with immunological phenotypes. **(A, B)** healthy control. **(C, D)** myasthenia gravis patient. **(E, F)** 22q11.2 microdeletion patient. **(A, C, E)** Left panel: flow cytometry of human thymus cell suspension with TECs gated as described. Right panel: HLA-DR fluorescence signal on mTECs (magenta) and cTECs (blue). **(E)** HLA-DR fluorescence signal on DCs (CD45+HLA-DR+CD11c+CD11b+) in grey for comparison. **(B, D, F)** Cryosections of human thymus. Nuclei are visualized with DAPI (blue), Cortex (c) and medulla (m) are distinguished based on density of nuclei (dashed line). No clear cortical or medullary areas in **(D)**. TECs are labelled with ck8 (yellow). Staining for ck13 in magenta. Scale bar 200 μm.

Supplementary Figure 7 Frequencies of the non-TEC stromal cells in pediatric thymi. Flow cytometry data of frequencies of human thymic stromal cell populations, grouped by the indicated age. All cells gated on live single cells. **(A)** Macrophages (MP) (CD45+HLA-DR+CD11c-CD11b+). **(B)** Dendritic cells (DC) (CD45+HLA-DR+CD11c+CD11b+). **(C)** Fibroblasts (FB) (EpCAM-pdpnintCD45-HLA-DR-). **(D)** Endothelial cells (EC) (CD31+pdpn-). **(E)** Lymphatic endothelial cells (LEC) (CD31+pdpn+).

## Abbreviations

ck, cytokeratin; cTEC, cortical thymic epithelial cell; DC, dendritic cell; EC, endothelial cell; ECM, extracellular matrix; FB, fibroblast; FC, flow cytometry; FPKM, fragments per kilobase of transcript per million mapped reads; FRC, fibroblastic reticular cell; ^high^, high expression level (flow cytometry); ^int^, intermediate expression level (flow cytometry); ^low^, low expression level (flow cytometry); MG, myasthenia gravis; MP, macrophage; mTEC, medullary thymic epithelial cell; pdpn, podoplanin; TEC, thymic epithelial cell; TRA, tissue restricted antigen; UEA-1, ulex europaeus agglutinin 1.
