# Supplementary figures and images for: Novel Combination of Surface Markers for the Reliable and Comprehensive Identification of Human Thymic Epithelial Cells by Flow Cytometry: Quantitation and Transcriptional Characterization of Thymic Stroma in a Pediatric Cohort
Source: Front Immunol. 2021 Sep 30;12:740047. doi: 10.3389/fimmu.2021.740047 (PMC8514761; doi:10.3389/fimmu.2021.740047)

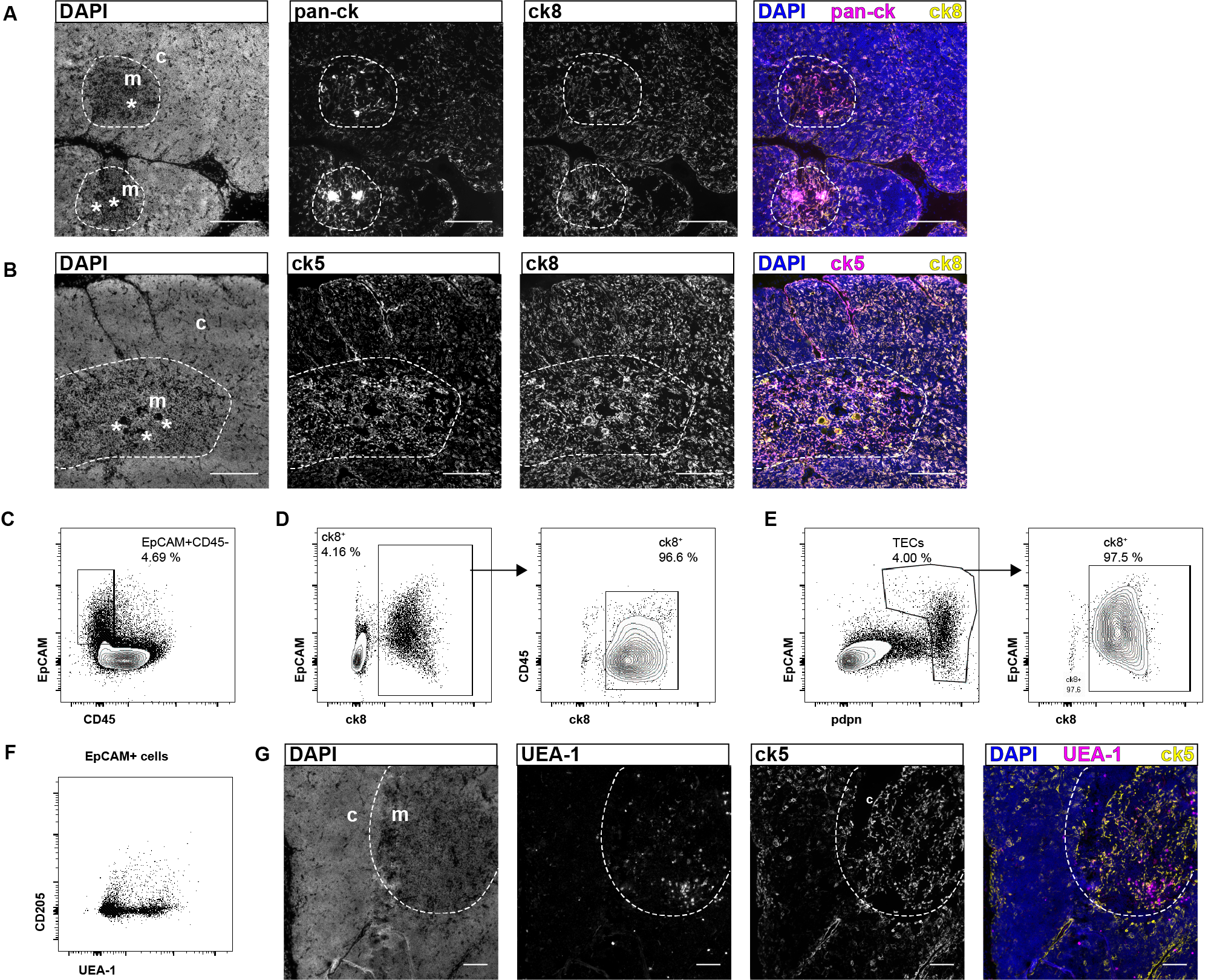

Supplement: Supplementary Figure 1 — Thymic epithelial cells can be defined by expression of ck8 and ck5, but not distinguished based on expression of CD205 or binding to UEA-1. (A, B, F) Cryosections of human thymus. Nuclei are visualized with DAPI (blue). Cortex (c) and medulla (m) are distinguished based on density of nuclei. Asterisks: Hassal’s corpuscules. (A) TECs are visualized with antibodies against pan-ck (magenta) and ck8 (yellow). Scale bar: 200 μm. (B) TECs are visualized with antibodies against ck5 (magenta) and ck8 (yellow). Scale bar: 200 μm. (C-E, G) Flow cytometry staining of APC-enriched human thymus cell suspension gated on live single cells. (C) Staining for EpCAM and CD45 (D) Left plot: Live single cells stained as shown in (C) Right plot: EpCAM and pdpn fluorescence signal of ck8+ cells. (E) Right plot: ck8 fluorescence signal of EpCAMhigh/int/lowpdpnhigh/int cells. (F) Flow cytometry staining of APC-enriched human thymus cell suspension for CD205 and UEA-1 gated on live single TECs (defined as EpCAMhigh/int/lowpdpnhigh/int). (G) Staining with UEA-1 (magenta). TECs are visualized with antibodies against ck5 (yellow). Scale bar: 100 μm. [file Image_1.tif]

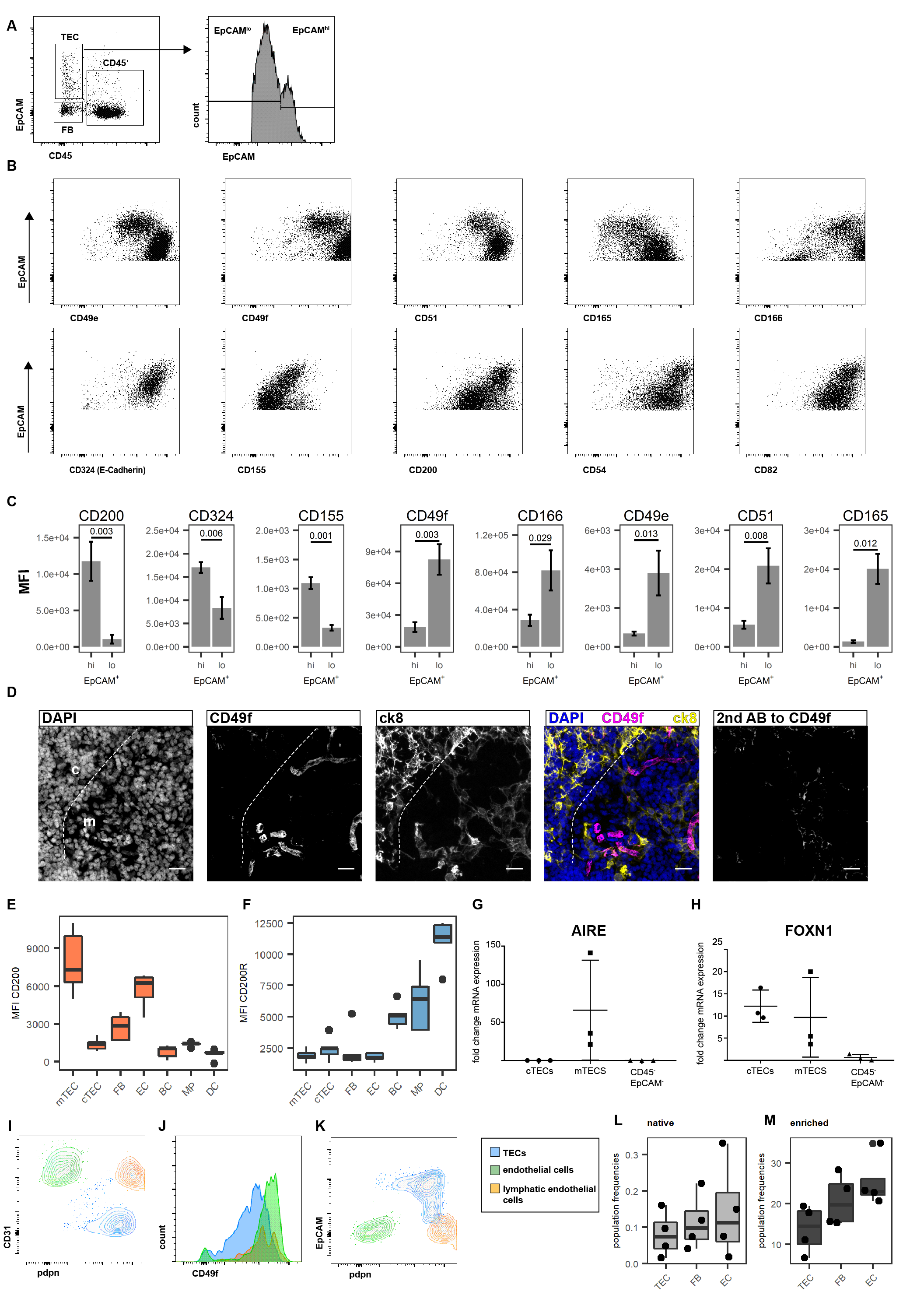

Supplement: Supplementary Figure 2 — Supplementary data on surface marker screen and cell sorting. (A) Left plot: Surface marker screen flow cytometry gating strategy to define TECs (CD45-EpCAM+), FB (CD45-EpCAM-) and CD45+ (CD45+) cells. Gated on single cells from partially CD45+ depleted cell suspension from human thymus. Right plot: Delineation of EpCAMhigh and EpCAMlow cells gated on TECs. (B) Individual flow cytometry plots of markers of interest from surface marker screen. Gated on TECs from (A). (C) Markers chosen for further evaluation and stained on four additional consecutive thymi. MFIs on CD45-EpCAMhigh and CD45-EpCAMlow cells are compared by paired t-test. (D) Cryosections of human thymus. Nuclei are visualized with DAPI (blue). Cortex (c) and medulla (m) are distinguished based on density of nuclei (dashed line). Staining for CD49f (magenta). TECs are visualized with antibodies against ck8 (yellow). Exposure time for CD49f was adapted according to the staining with secondary antibody to CD49f (left panel). Scale bar 50 μm. (E, F) Mean fluorescence intensity (MFI) of CD200 (E) and CD200R (F) on thymic cell populations. Gated on live single cells. MP, macrophages; DC, dendritic cells. (G, H) Relative mRNA expression of cell-sorted putative cTECs, mTECs and CD45-EpCAM- cells. Fold change is calculated relative to the median expression level of all samples. (G) AIRE mRNA expression. (H) FOXN1 mRNA expression. (I-K) Fluorescence signal (flow cytometry) of TECs (EpCAMhigh/int/lowpdpnhigh/int, blue), endothelial cells (CD31+pdpn-, green) and lymphatic endothelial cells (CD31+pdpn+, orange) for CD31 (I), CD49f (J) or EpCAM and pdpn (K). (L, M) Frequency of TEC, FB and EC (% of live cells) in cell suspensions (digest) prior to (L) and after percoll-density- and CD45-bead-based enrichment (M). [file Image_2.tif]

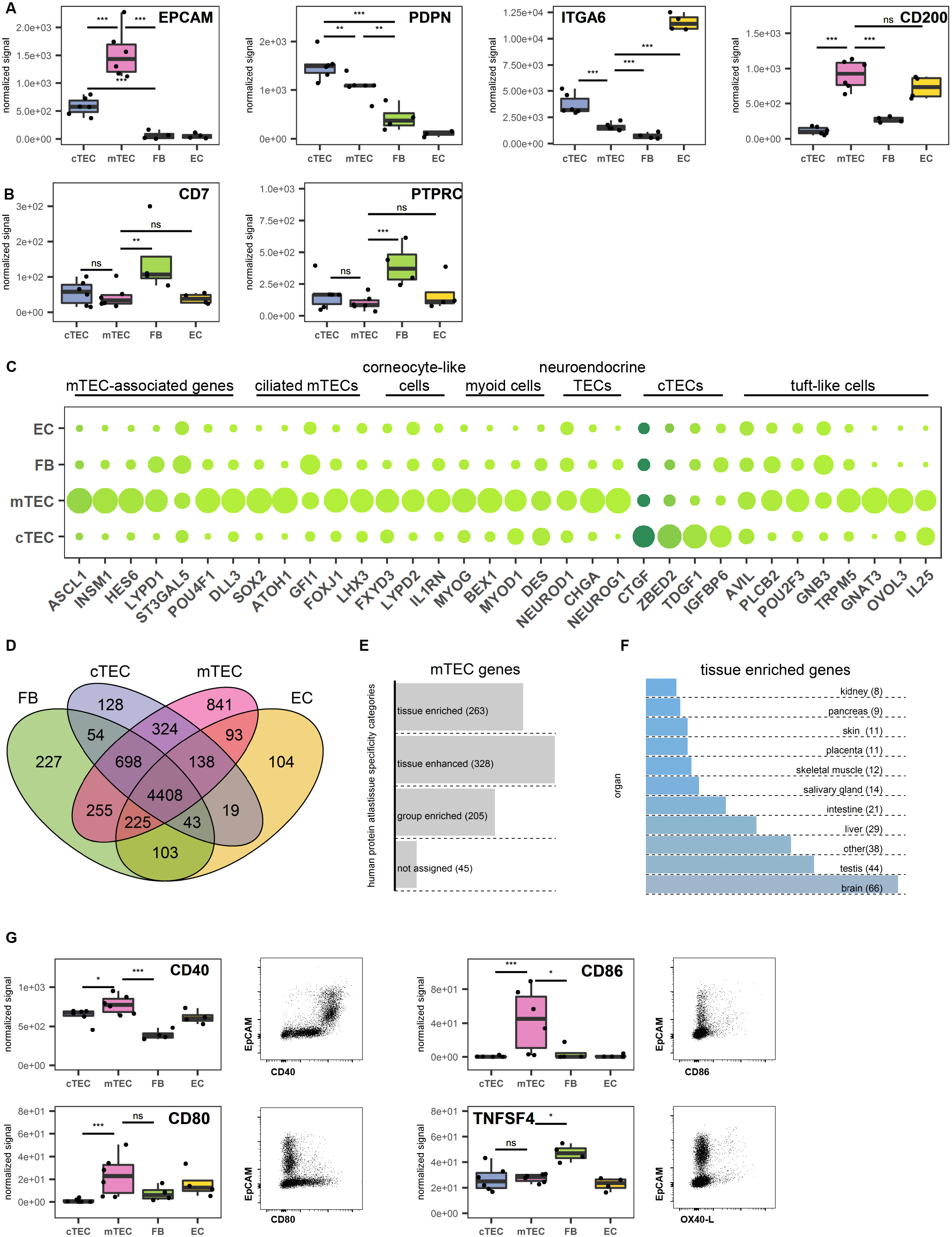

Supplement: Supplementary Figure 3 — Validation of the flow cytometry gating strategy and expression of costimulatory molecules. (A, B) boxplot representation of genes from the transcriptomic dataset. ***p < 0.001, **p < 0.01, *p < 0.05, ns, not significant. (A) Genes coding for markers used in the sorting strategy. (B) Genes characteristic for thymocytes or hematopoietic cells. (C) Genes associated with TEC subpopulations according to the sorted cell population. Colour gradient reflects the mean expression signal for each gene across all cell populations, dot size represents the relative expression value of each gene between cell populations. Genes are grouped in categories as labelled. (D) Genes that are expressed in at least 3 out of 6 (mTEC and cTEC populations) or 2 out of 4 (FB and EC populations) at FPKM >0.5. (E) Genes from (D) exclusively expressed in mTECs compared to the human protein atlas tissue specificity categories. The number of matched genes for each category is given in brackets. (F) Genes from the tissue enriched category in (E) are plotted according to their organ. Number of matching genes for each organ is given in brackets. (G) Result for co-stimulatory molecules in transcriptomic dataset (left column) and CD45- cells (gated on single cells) from the flow cytometry surface marker screen (right column). Boxplots: ***p < 0.001, *p < 0.05, ns, p > 0.05. ns, not significant. [file Image_3.tif]

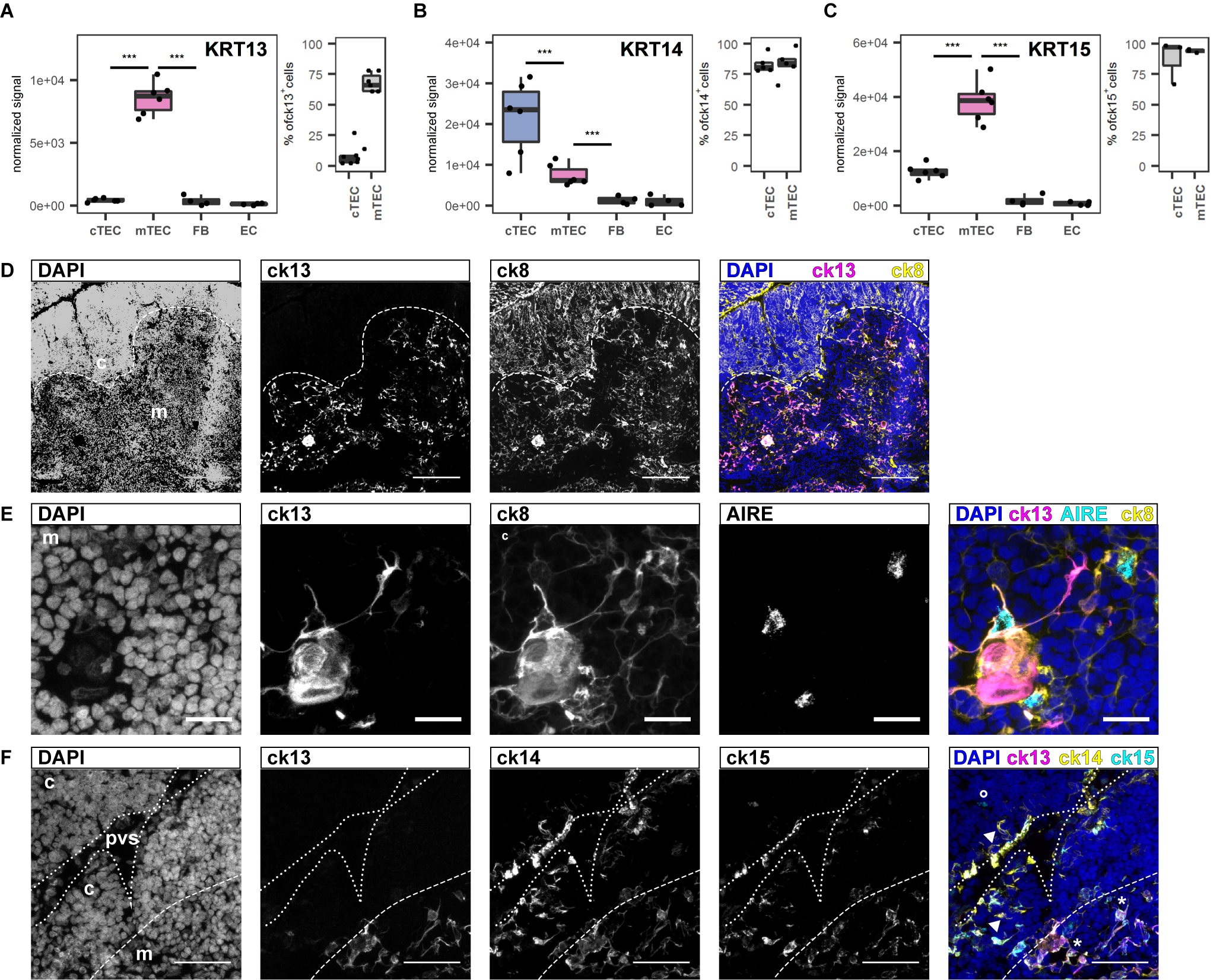

Supplement: Supplementary Figure 4 — Cytokeratins as markers for human TEC subsets. (A-C) left panel: Gene expression data from the transcriptomic dataset for KRT13 (A), KRT14 (B) and KRT15 (C). ***p < 0.001. (A-C) right panel: flow cytometry analysis of cytokeratin 13 (ck13), 14 (ck14) and 15 (ck15) on 5 thymi (ck13, ck14) or 3 thymi (ck15). cTECs and mTECs were gated according to the proposed gating strategy. (D-F) Cryosections of human thymus. Nuclei are visualized with DAPI (blue). Cortex (c) and medulla (m) are distinguished based on density of nuclei (dashed line). Perivascular space (pvs) indicated by dotted line. (D) Staining for ck13 (magenta). TECs are visualized with ck8 (yellow). Scale bar 200 μm. (E) High resolution image of thymic medulla. Staining for ck13 (magenta), staining for AIRE (cyan). TECs are visualized with ck8 (yellow). Scale bar 20 μm. (F) Staining for ck13 (magenta), ck14 (yellow) and ck15 (cyan). Scale bar 100 μm. Asterisk: ck13+ck14+ck15+ cell. Arrowhead: ck14+ cell. Circle: ck15+ cell. [file Image_4.tif]

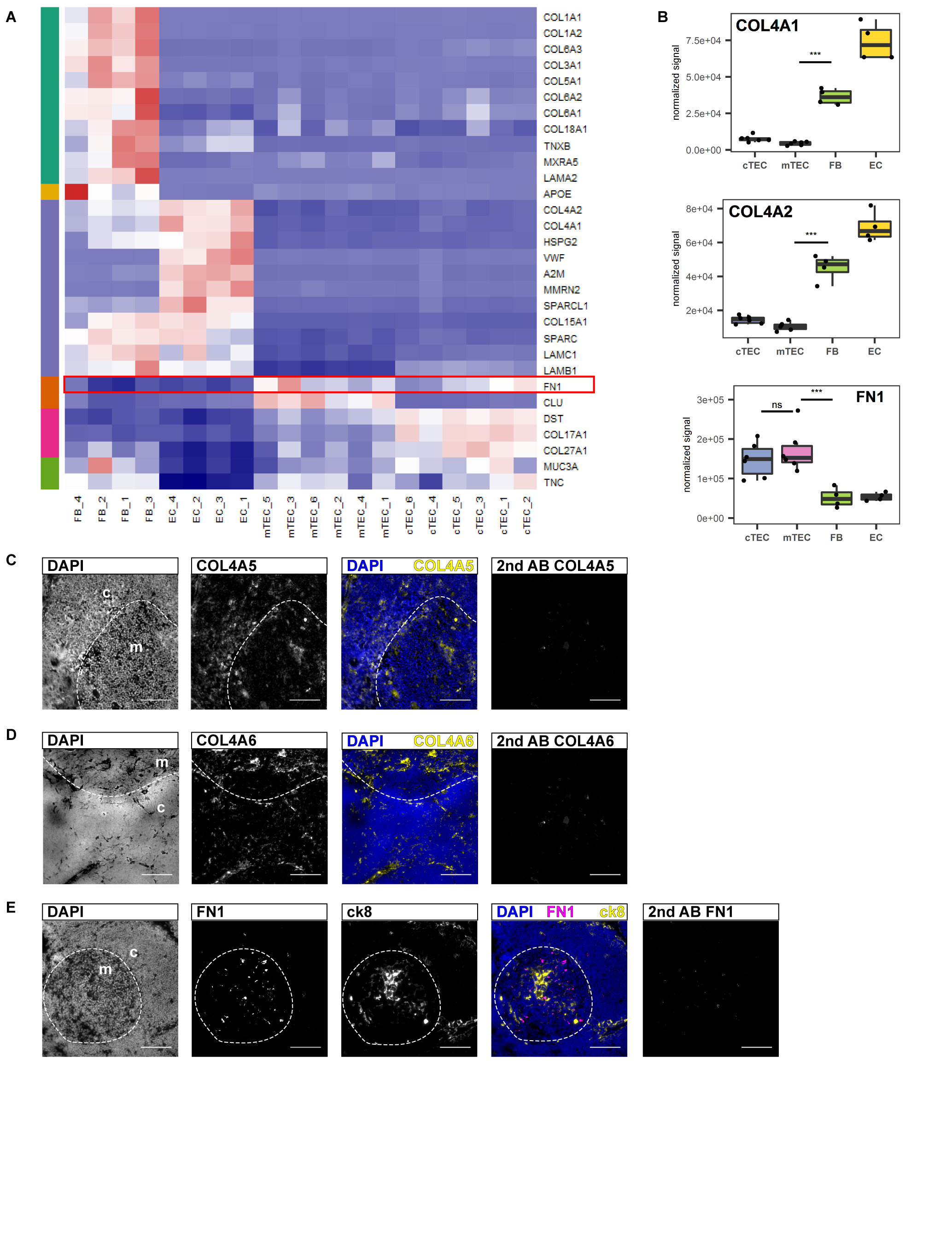

Supplement: Supplementary Figure 5 — Supplementary information for gene expression profile of ECM components. (A) Heat map of the 30 genes with highest variance between all four cell populations that are associated with the GO term “extracellular matrix”. (B) Gene expression data for COL4A1, COL4A2 and FN1. ***p< 0.01, ns: not significant. (C-E) Cryosections of human thymus. Nuclei in blue. Cortex (c) and medulla (m) are distinguished based on density of nuclei (dashed line). Exposure time was adapted according to staining with secondary antibody. (C) Staining for Collagen IV α5 (yellow). Scale bar 200 μm. (D) Staining for Collagen IV α6 (yellow). Scale bar 200 μm. (E) Staining for FN1 (magenta). TECs are visualized with ck8 (yellow). (E) Scale bar 200 μm. [file Image_5.tif]

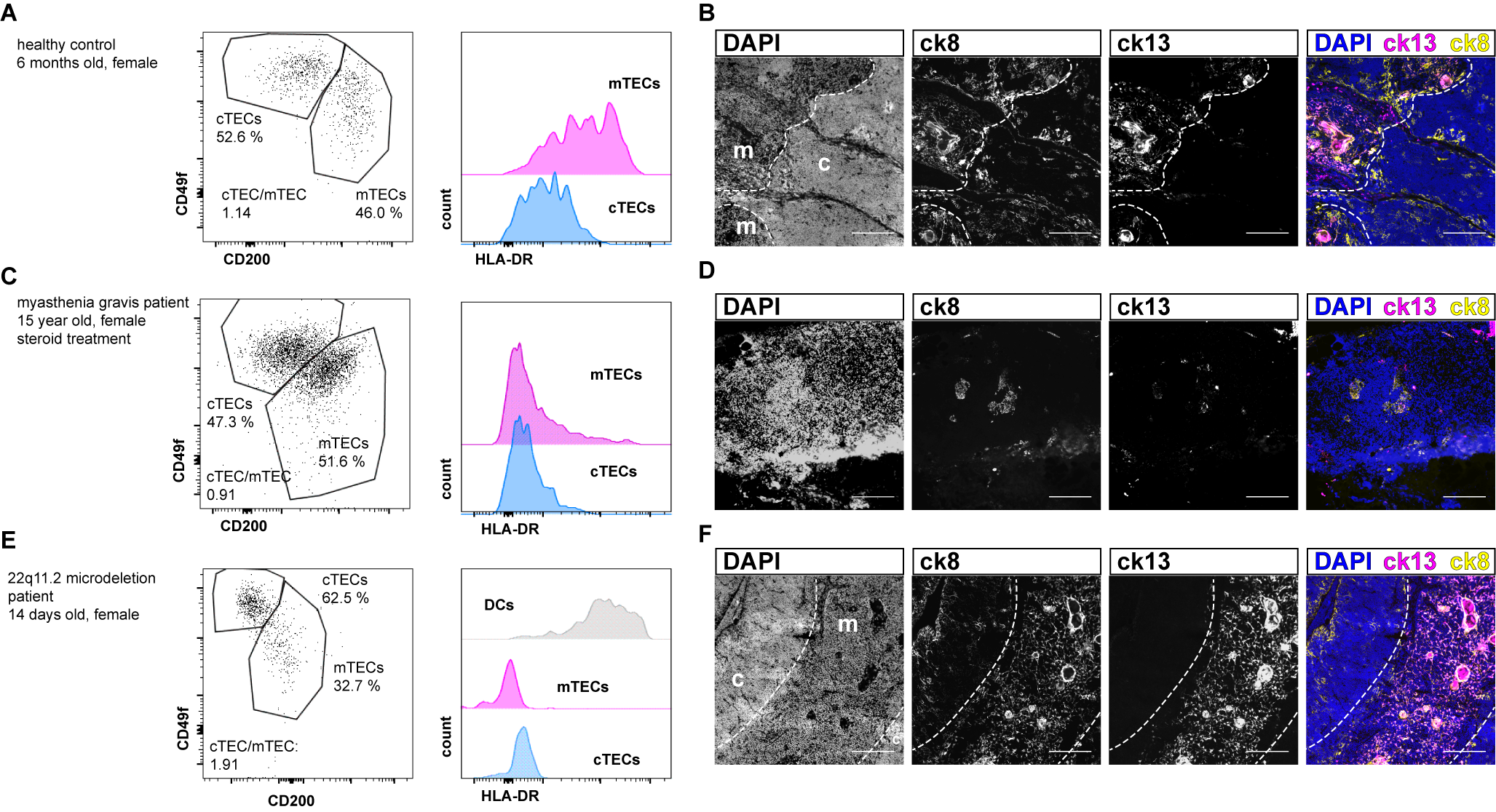

Supplement: Supplementary Figure 6 — Analysis of thymus tissue from patients with immunological phenotypes. (A, B) healthy control. (C, D) myasthenia gravis patient. (E, F) 22q11.2 microdeletion patient. (A, C, E) Left panel: flow cytometry of human thymus cell suspension with TECs gated as described. Right panel: HLA-DR fluorescence signal on mTECs (magenta) and cTECs (blue). (E) HLA-DR fluorescence signal on DCs (CD45+HLA-DR+CD11c+CD11b+) in grey for comparison. (B, D, F) Cryosections of human thymus. Nuclei are visualized with DAPI (blue), Cortex (c) and medulla (m) are distinguished based on density of nuclei (dashed line). No clear cortical or medullary areas in (D). TECs are labelled with ck8 (yellow). Staining for ck13 in magenta. Scale bar 200 μm. [file Image_6.tif]

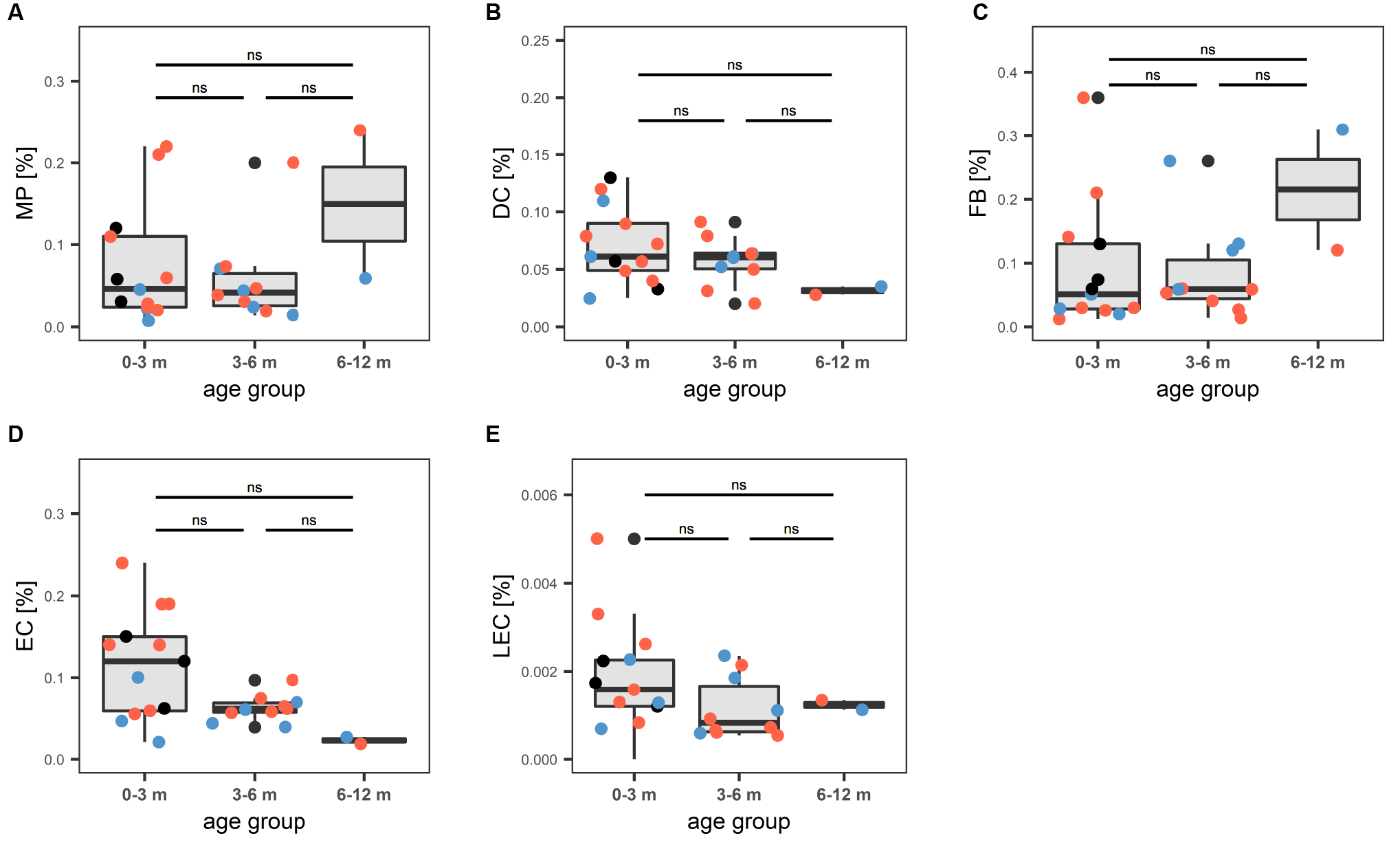

Supplement: Supplementary Figure 7 — Frequencies of the non-TEC stromal cells in pediatric thymi. Flow cytometry data of frequencies of human thymic stromal cell populations, grouped by the indicated age. All cells gated on live single cells. (A) Macrophages (MP) (CD45+HLA-DR+CD11c-CD11b+). (B) Dendritic cells (DC) (CD45+HLA-DR+CD11c+CD11b+). (C) Fibroblasts (FB) (EpCAM-pdpnintCD45-HLA-DR-). (D) Endothelial cells (EC) (CD31+pdpn-). (E) Lymphatic endothelial cells (LEC) (CD31+pdpn+). [file Image_7.tif]
