# Supplementary material for: Novel Combination of Surface Markers for the Reliable and Comprehensive Identification of Human Thymic Epithelial Cells by Flow Cytometry: Quantitation and Transcriptional Characterization of Thymic Stroma in a Pediatric Cohort
Source: Front Immunol. 2021 Sep 30;12:740047. doi: 10.3389/fimmu.2021.740047 (PMC8514761; doi:10.3389/fimmu.2021.740047)
Supplement: Supplementary file 8 [file Table_1.docx]

# S1 Table. Markers with biological significance for mouse and human TEC biology

| **marker** | **function** | **mouse** | **human** | **detection method (human)** |
| --- | --- | --- | --- | --- |
| CLDN3 | tight junction protein | ^1^ | ^2^ | IF-Fr, IF culture |
| CLDN4 | tight junction protein | ^1^ | ^3,4^ | IF-Fr, IF culture |
| TNFRSF11A/RANK | Thymic crosstalk/medulla formation | ^5–7^ | ^8^ | scRNA |
| AIRE | Transcriptional activator/TRA expression | ^9–11^ | ^12–14^ | IHC, in situ hybridization, RNA |
| FEZF2 | AIRE-independent TRA expression | ^15^ | ^2,15^ | IF-Fr, RT-PCR |
| TNFS4/OX40-L | Costimulation/thymic crosstalk/Treg induction | ^16^ | ^16^ | FC |
| CD80 | Costimulation/thymic crosstalk/Treg induction | ^17^ |  |  |
| CD86 | Costimulation/thymic crosstalk/Treg induction | ^17^ |  |  |
| CD40 | Costimulation/thymic crosstalk/Treg induction | ^6,17,18^ | ^19^ | FC, IF-Fr |
| CCL25 | Cortical retention signal | ^20^ | ^4^ | IF-Fr |
| PRDM1 | Transcription factor important for mTEC function | ^21^ | ^2,21^ | IHC, RT-PCR culture |
| PSMB11 | Thymoproteasome catalytic subunit β5t | ^22^ | ^23^ | RT-PCR, IF-Fr |
| PRSS16 | Thymus-specific serine protease (TSSP); proteolytic cleavage of proteins | ^24^ | ^25^ | Northern Blot, IF-Fr |
| CTSV | Cathepsin V; proteolytic cleavage of proteins | ^26,27^ | ^28^ | RT-PCR, IHC |
| RELB | NFκB family member; mTEC signaling | ^29,30^ | ^31^ | IHC |
| TP63 | Transcription factor; mTEC signaling |  | ^3,32^ | IF-Fr, IF culture |
| DLL1 | Thymocyte survival | ^33^ | ^34^ | IF-Fr |
| DLL4 | thymocyte lineage commitment | ^33,35^ | ^34^ | IF-Fr |
| CCL19 | Medullary migration signal; ligand to CCR7 | ^36^ |  |  |
| CCL21 | Medullary migration signal; ligand to CCR7 | ^20^ | ^4^ | IF-Fr |
| FOXN1 | Transcription factor involved in onset of thymogenesis/TEC differentiation | ^37^ | ^4,38^ | IHC |
|  |  |  |  |  |
| **marker** | **function** | **mouse** | **human** | **detection method (human)** |
|  |  |  |  |  |
| PAX1 | Transcription factor involved in thymogenesis | ^39^ ^40^ | ^4,41^ | functional studies, IF-Fr |
| PAX3 | Transcription factor involved in thymogenesis | ^42^ |  |  |
| PAX9 | Transcription factor involved in thymogenesis | ^43^ | ^4^ | IF-Fr |
| TBX1 | Transcription factor involved in thymogenesis; responsible for thymic phenotype of DiGeorge/22q11.2 microdeletion | ^44^ | ^4^ | IF-Fr |
| SIX1 | Transcription factor involved in thymogenesis | ^45^ |  |  |
| EYA1 | Transcription factor involved in thymogenesis | ^46^ |  |  |
| HOXA3 | Transcription factor involved in thymogenesis | ^40^ | ^4^ | IF-Fr |
| IVL | Involucrin; marker for mTEC maturation |  | ^47^ | IHC |
| LTBR | Lymphotoxin β receptor; thymic crosstalk | ^48–50^ | ^51^ | IF-Fr |
| TSLP | Thymic stromal lymphopoietin; involved in Treg formation | ^52,53^ | ^54,55^ | IHC |
| ICOSLG | ICOS-Ligand; involved in Treg formation | ^56^ | ^57^ | IF culture |
| IL7 | Thymocyte survival signal | ^58,59^ |  |  |
| ACKR4 | CCRL1; alternative ligand for CCR7 (?) | ^60^ |  |  |
| HDAC3 | Transcriptional regulator of mTEC differentiation/maturation | ^61^ |  |  |
| SIRT1 | Required for expression of AIRE-dependent TRAs | ^62^ | ^62^ | co-IP with AIRE (HEK293 cells) |
| DLK2 | NOTCH homologue involved in morphogenesis | ^63^ | ^88^ | scRNA |
| ASCL1 | Upreglation upon HDAC3 activation; involved in mTEC maturation | ^61^ |  |  |
| POU2F3 | Transcription factor characteristic of mTEC subset | ^64^ |  |  |
| EHF | Upreglation upon HDAC3 activation; involved in mTEC maturation | ^61^ |  |  |
|  |  |  |  |  |
| **marker** | **function** | **mouse** | **human** | **detection method (human)** |
|  |  |  |  |  |
| SPIB | Upreglation upon HDAC3 activation; involved in mTEC maturation | ^61,65^ |  |  |
| CREBBP | Transcriptional co-activator; cooperates with AIRE | ^66^ |  |  |
| FGFR2 | TEC development/maintenance; signals from mesenchymal cells | ^67^ |  |  |
|  |  |  |  |  |
| other markers |  |  |  |  |
|  |  |  |  |  |
| UEA1 | Marks thymic medulla | ^68^ | ^2,42,4^ | IF-Fr, IF culture |
| CDR2 | Clone with unknown binding epitope; marks cTECs |  | ^69^ | IHC |

## Abbreviations:

IF-Fr Immunofluorescence frozen sections

IF culture Immunofluorescence cell cultures

IHC Immunohistochemistry

scRNA single cell sequencing RNA

FC flow cytometry

co-IP co-immunoprecipitation

# References for Supplementary Table 1:

1. Hamazaki Y, Fujita H, Kobayashi T, et al. Medullary thymic epithelial cells expressing Aire represent a unique lineage derived from cells expressing claudin. *Nat Immunol*. 2007;8(3):304-311.

2. Villegas JA, Gradolatto A, Truffault F, et al. Cultured human thymic-derived cells display medullary thymic epithelial cell phenotype and functionality. *Front Immunol*. 2018;9(JUL):1-12.

3. Ichimiya S, Kojima T. Cellular Networks of Human Thymic Medullary Stromas Coordinated by p53-Related Transcription Factors. *J Histochem Cytochem*. 2006;54(11):1277-1289. doi:10.1369/jhc.6A7028.2006.

4. Farley AM, Morris LX, Vroegindeweij E, et al. Dynamics of thymus organogenesis and colonization in early human development. *Development*. 2013;140(9):2015-2026.

5. Rossi SW, Kim MY, Leibbrandt A, et al. RANK signals from CD4+3- inducer cells regulate development of Aire-expressing epithelial cells in the thymic medulla. *J Exp Med*. 2007;204(6):1267-1272.

6. Akiyama T, Shimo Y, Yanai H, et al. The Tumor Necrosis Factor Family Receptors RANK and CD40 Cooperatively Establish the Thymic Medullary Microenvironment and Self-Tolerance. *Immunity*. 2008;29(3):423-437.

7. Hikosaka Y, Nitta T, Ohigashi I, et al. The Cytokine RANKL Produced by Positively Selected Thymocytes Fosters Medullary Thymic Epithelial Cells that Express Autoimmune Regulator. *Immunity*. 2008;29(3):438-450.

8. Park JE, Botting RA, Conde CD, et al. A cell atlas of human thymic development defines T cell repertoire formation. *Science (80- )*. 2020;367(6480).

9. Blechschmidt K, Schweiger M, Wertz K, et al. The mouse Aire gene: Comparative genomic sequencing, gene organization, and expression. *Genome Res*. 1999;9(2):158-166.

10. Ruan QG, Wang CY, Shi JD, She JX. Expression and alternative splicing of the mouse autoimmune regulator gene (Aire). *J Autoimmun*. 1999;13(3):307-313.

11. Mittaz L, Rossier C, Heino M, et al. Isolation and characterization of the mouse Aire gene. *Biochem Biophys Res Commun*. 1999;255(2):483-490.

12. Nagamine K, Peterson P, Scott HS, et al. Positional cloning of the APECED gene. *Nat Genet*. 1997;17(4):393-398.

13. Björses P, Pelto-Huikko M, Kaukonen J, Aaltonen J, Peltonen L, Ulmanen I. Localization of the APECED protein in distinct nuclear structures. *Hum Mol Genet*. 1999;8(2):259-266.

14. Heino M, Peterson P, Kudoh J, et al. Autoimmune regulator is expressed in the cells regulating immune tolerance in thymus medulla. *Biochem Biophys Res Commun*. 1999;257(3):821-825.

15. Takaba H, Morishita Y, Tomofuji Y, et al. Fezf2 Orchestrates a Thymic Program of Self-Antigen Expression for Immune Tolerance. *Cell*. 2015;163(4):975-987.

16. Kumar P, Marinelarena A, Raghunathan D, et al. Critical role of OX40 signaling in the TCR-independent phase of human and murine thymic Treg generation. *Cell Mol Immunol*. 2019;16(2):138-153.

17. Williams JA, Zhang J, Jeon H, et al. Thymic Medullary Epithelium and Thymocyte Self-Tolerance Require Cooperation between CD28–CD80/86 and CD40–CD40L Costimulatory Pathways. *J Immunol*. 2014;192(2):630-640.

18. Gray DHD, Seach N, Ueno T, et al. Developmental kinetics, turnover, and stimulatory capacity of thymic epithelial cells. *Blood*. 2006;108(12):3777-3785.

19. Galy A, Spits H. CD40 is functionally expressed on human thymic epithelial cells. *J Immunol*. 1992;149:775-782.

20. Liu C, Saito F, Liu Z, et al. Coordination between CCR7- and CCR9-mediated chemokine signals in prevascular fetal thymus colonization. *Blood*. 2006;108(8):2531-2539.

21. Roberts NA, Adams BD, McCarthy NI, et al. Prdm1 Regulates Thymic Epithelial Function To Prevent Autoimmunity. *J Immunol*. 2017;199(4):1250-1260. doi:10.4049/jimmunol.1600941.

22. Murata S, Sasaki K, Kishimoto T, et al. Regulation of CD8+ T Cell Development by Thymus-Specific Proteasomes. 2007;316(June):1349-1354.

23. Tomaru U, Ishizu A, Murata S, et al. Exclusive expression of proteasome subunit β5t in the human thymic cortex. *Blood*. 2009;113(21):5186-5191. doi:10.1182/blood-2008-11-187633.

24. Carrier A, Nguyen C, Victorero G, et al. Differential gene expression in CD3ε and RAG1-deficient thymuses: Definition of a set of genes potentially involved in thymocyte maturation. *Immunogenetics*. 1999;50(5-6):255-270.

25. Bowlus CL, Ahn J, Chu T, Gruen JR. Cloning of a novel MHC-encoded serine peptidase highly expressed by cortical epithelial cells of the thymus. *Cell Immunol*. 1999;196(2):80-86. doi:10.1006/cimm.1999.1543.

26. Nakagawa T, Roth W, Wong P, et al. Cathepsin L: Critical role in Ii degradation and CD4 T cell selection in the thymus. *Science (80- )*. 1998;280(5362):450-453.

27. Honey K, Nakagawa T, Peters C, Rudensky A. Cathepsin L regulates CD4+ T cell selection independently of its effect on invariant chain: A role in the generation of positively selecting peptide ligands. *J Exp Med*. 2002;195(10):1349-1358.

28. Tolosa E, Li W, Yasuda Y, et al. Cathepsin V is involved in the degradation of invariant chain in human thymus and is overexpressed in myasthenia gravis. *J Clin Invest*. 2003;112(4):517-526. doi:10.1172/JCI200318028.

29. Burkly L, Hession C, Ogata L, et al. Expression of relB is required for the development of thymic medulla and dendritic cells. *Nature*. 1995;373(6514):531-536.

30. Baik S, Sekai M, Hamazaki Y, Jenkinson WE, Anderson G. Relb acts downstream of medullary thymic epithelial stem cells and is essential for the emergence of RANK+ medullary epithelial progenitors. *Eur J Immunol*. 2016;46(4):857-862.

31. Thompson AG, Pettit AR, Padmanabha J, et al. Nuclear RelB+ cells are found in normal lymphoid organs and in peripheral tissue in the context of inflammation, but not under normal resting conditions. *Immunol Cell Biol*. 2002;80(2):164-169. doi:10.1046/j.1440-1711.2002.01070.x.

32. Kikuchi T, Ichimiya S, Kojima T, et al. Expression profiles and functional implications of p53-like transcription factors in thymic epithelial cell subtypes. *Int Immunol*. 2004;16(6):831-841.

33. Hozumi K, Negishi N, Suzuki D, et al. Delta-like 1 is necessary for the generation of marginal zone B cells but not T cells in vivo. *Nat Immunol*. 2004;5(6):638-644.

34. García-León MJ, Fuentes P, de la Pompa JL, Toribio ML. Dynamic regulation of NOTCH1 activation and notch ligand expression in human thymus development. *Dev*. 2018;145(16 Special Issue).

35. Hozumi K, Mailhos C, Negishi N, et al. Delta-like 4 is indispensable in thymic environment specific for T cell development. *J Exp Med*. 2008;205(11):2507-2513.

36. Ueno T, Saito F, Gray DHD, et al. CCR7 signals are essential for cortex-medulla migration of developing thymocytes. *J Exp Med*. 2004;200(4):493-505.

37. Nehls M, Pfeifer D, Schorpp M, Hedrich H, Boehm T. New member of the winged-helix protein family disrupted in mouse and rat nude mutations. 1994;372:103-107.

38. Pignata C, Fiore M, Guzzetta V, et al. Congenital alopecia and nail dystrophy associated with severe functional T-cell immunodeficiency in two sibs. *Am J Med Genet*. 1996;65(2):167-170.

39. Wallin J, Eibel H, Neubüser A, Wilting J, Koseki H, Balling R. Pax1 is expressed during development of the thymus epithelium and is required for normal T-cell maturation. *Development*. 1996;122(1):23-30.

40. Su D, Manley NR. Hoxa3 and Pax1 Transcription Factors Regulate the Ability of Fetal Thymic Epithelial Cells to Promote Thymocyte Development. *J Immunol*. 2000;164(11):5753-5760.

41. Yamazaki Y, Urrutia R, Franco LM, et al. PAX1 is essential for development and function of the human thymus. *Sci Immunol*. 2020;5(44). doi:10.1126/sciimmunol.aax1036.

42. Conway SJ, Henderson DJ, Copp AJ. Pax3 is required for cardiac neural crest migration in the mouse: Evidence from the splotch (Sp(2H)) mutant. *Development*. 1997;124(2):505-514.

43. Peters H, Neubüser A, Kratochwil K, Balling R. Pax9-deficient mice lack pharyngeal pouch derivatives and teeth and exhibit craniofacial and limb abnormalities. *Genes Dev*. 1998;12(17):2735-2747.

44. Jerome LA, Papaioannou VE. DiGeorge syndrome phenotype in mice mutant for the T-box gene, Tbx1. *Nat Genet*. 2001;27(3):286-291.

45. Laclef C, Souil E, Demignon J, Maire P. Thymus, kidney and craniofacial abnormalities in Six1 deficient mice. *Mech Dev*. 2003;120(6):669-679.

46. Xu P-X, Zheng W, Laclef C, et al. Eya1 is required for the morphogenesis of mammalian thymus, parathyroid and thyroid. *Development*. 2002;129(13):3033-3044.

47. Bodey B, Bodey BJ, Siegel SE, Kaiser HE. Novel insights into the function of the thymic Hassall’s bodies. *In Vivo (Brooklyn)*. 2000;14(3):407-418.

48. Lkhagvasuren E, Sakata M, Ohigashi I, Takahama Y. Lymphotoxin β Receptor Regulates the Development of CCL21-Expressing Subset of Postnatal Medullary Thymic Epithelial Cells. *J Immunol*. 2013;190(10):5110-5117.

49. Venanzi ES, Gray DHD, Benoist C, Mathis D. Lymphotoxin Pathway and Aire Influences on Thymic Medullary Epithelial Cells Are Unconnected. *J Immunol*. 2007;179(9):5693-5700.

50. White AJ, Nakamura K, Jenkinson WE, et al. Lymphotoxin Signals from Positively Selected Thymocytes Regulate the Terminal Differentiation of Medullary Thymic Epithelial Cells. *J Immunol*. 2010;185(8):4769-4776.

51. Murphy M, Walter BN, Pike-Nobile L, et al. Expression of the lymphotoxin β receptor on follicular stromal cells in human lymphoid tissues. *Cell Death Differ*. 1998;5(6):497-505.

52. Park LS, Martin U, Garka K, et al. Cloning of the murine thymic stromal lymphopoietin (TSLP) receptor: Formation of a functional heteromeric complex requires interleukin 7 receptor. *J Exp Med*. 2000;192(5):659-669.

53. Sims JE, Williams DE, Morrissey PJ, et al. Molecular cloning and biological characterization of a novel murine lymphoid growth factor. *J Exp Med*. 2000;192(5):671-680.

54. Watanabe N, Wang Y-H, Lee HK, et al. Hassall’s corpuscles instruct dendritic cells to induce CD4+CD25+ regulatory T cells in human thymus. *Nature*. 2005;436(August):1181-1185.

55. Friend SL, Hosier S, Nelson A, Foxworthe D, Williams DE, Farr A. A thymic stromal cell line supports in vitro development of surface IgM+ B cells and produces a novel growth factor affecting B and T lineage cells. *Exp Hematol*. 1994;22(3):321-328.

56. White AJ, Jenkinson WE, Cowan JE, et al. An Essential Role for Medullary Thymic Epithelial Cells during the Intrathymic Development of Invariant NKT Cells. *J Immunol*. 2014;192(6):2659-2666.

57. Nazzal D, Gradolatto A, Truffault F, Bismuth J, Berrih-Aknin S. Human thymus medullary epithelial cells promote regulatory T-cell generation by stimulating interleukin-2 production via ICOS ligand. *Cell Death Dis*. 2014;5(9):e1420.

58. Moore NC, Anderson G, Smith CA, Owen JJ, Jenkinson EJ. Analysis of cytokine gene expression in subpopulations of freshly isolated thymocytes and thymic stromal cells using semiquantitative polymerase chain reaction. *Eur J Immunol*. 1993;23(4):922-927.

59. Murray R, Suda T, Wrighton N, Lee F, Ziotnik A. IL-7 is a growth and maintenance factor for mature and immature thymocyte subsets. *Int Immunol*. 1989;1(5):526-531.

60. Lucas B, White AJ, Ulvmar MH, et al. CCRL1/ACKR4 is expressed in key thymic microenvironments but is dispensable for T lymphopoiesis at steady state in adult mice. *Eur J Immunol*. 2015;45(2):574-583.

61. Goldfarb Y, Kadouri N, Levi B, et al. HDAC3 Is a Master Regulator of mTEC Development. *Cell Rep*. 2016;15(3):651-665.

62. Chuprin A, Avin A, Goldfarb Y, et al. The deacetylase Sirt1 is an essential regulator of Aire-mediated induction of central immunological tolerance. *Nat Immunol*. 2015;16(7):737-745.

63. Garcia-Gallastegi P, Ruiz-García A, Ibarretxe G, et al. Similarities and differences in tissue distribution of DLK1 and DLK2 during E16.5 mouse embryogenesis. *Histochem Cell Biol*. 2019;152(1):47-60.

64. Bornstein C, Nevo S, Giladi A, et al. Single-cell mapping of the thymic stroma identifies IL-25-producing tuft epithelial cells. *Nature*. 2018;559(7715):622-626.

65. Kernfeld EM, Genga RM, Neherin K, Magaletta ME, Xu P, Maehr R. A single-cell transcriptomic atlas of thymus organogenesis resolves cell types and developmental maturation. *Immunity*. 2018;48(6):1258-1270.

66. Saare M, Rebane A, Rajashekar B, Vilo J, Peterson P. Autoimmune regulator is acetylated by transcription coactivator CBP/p300. *Exp Cell Res*. 2012;318(14):1767-1778.

67. Revest J-M, Suniara RK, Kerr K, Owen JJT, Dickson C. Development of the Thymus Requires Signaling Through the Fibroblast Growth Factor Receptor R2-IIIb. *J Immunol*. 2001;167(4):1954-1961.

68. Farr AG, Anderson SK. Epithelial heterogeneity in the murine thymus : fucose-specific lectins bind medullary epithelial cells. 2018.

69. Rouse R V., Bolin LM, Bender JR, Kyewski BA. Monoclonal antibodies reactive with subsets of mouse and human thymic epithelial cells. *J Histochem Cytochem*. 1988;36(12):1511-1517.
